# Evaluation of the Current Therapeutic Approaches for COVID-19: A Systematic Review and a Meta-analysis

**DOI:** 10.3389/fphar.2021.607408

**Published:** 2021-03-15

**Authors:** Zeinab Abdelrahman, Qian Liu, Shanmei Jiang, Mengyuan Li, Qingrong Sun, Yue Zhang, Xiaosheng Wang

**Affiliations:** ^1^ Biomedical Informatics Research Lab, School of Basic Medicine and Clinical Pharmacy, China Pharmaceutical University, Nanjing, China; ^2^ Big Data Research Institute, China Pharmaceutical University, Nanjing, China; ^3^ Pinghu Hospital of Shenzhen University, Shenzhen, China; ^4^ Futian Hospital for Rheumatic Diseases, Shenzhen, China; ^5^ Department of Rheumatology and Immunology, The First Clinical College of Harbin Medical University, Harbin, China

**Keywords:** COVID-19 treatment, meta-analysis, antiviral agents, hydroxychloroquine, dexamethasone, remdesivir

## Abstract

**Background:** Limited data on the efficacy and safety of currently applied COVID-19 therapeutics and their impact on COVID-19 outcomes have raised additional concern.

**Objective and Methods:** To estimate the efficacy and safety of COVID-19 therapeutics, we performed meta-analyses of the studies reporting clinical features and treatments of COVID-19 published from January 21 to September 6, 2020.

**Results:** We included 136 studies that involved 102,345 COVID-19 patients. The most prevalent treatments were antibiotics (proportion: 0.59, 95% CI: [0.51, 0.67]) and antivirals (proportion: 0.52, 95% CI: [0.44, 0.60]). The combination of lopinavir/ritonavir and Arbidol was the most effective in treating COVID-19 (standardized mean difference (SMD) = 0.68, 95% CI: [0.15, 1.21]). The use of corticosteroids was associated with a small clinical improvement (SMD = −0.40, 95% CI: [−0.85, −0.23]), but with a higher risk of disease progression and death (mortality: RR = 9.26, 95% CI: [4.81, 17.80]; hospitalization length: RR = 1.54, 95% CI: [1.39, 1.72]; severe adverse events: RR = 2.65, 95% CI: [2.09, 3.37]). The use of hydroxychloroquine was associated with a higher risk of death (RR = 1.68, 95% CI: [1.18, 2.38]). The combination of lopinavir/ritonavir, ribavirin, and interferon-β (RR = 0.34, 95% CI: [0.22, 0.54]); hydroxychloroquine (RR = 0.58, 95% CI: [0.39, 0.58]); and lopinavir/ritonavir (RR = 0.72, 95% CI: [0.56, 0.91]) was associated with reduced hospitalization length. Hydrocortisone (RR = 0.05, 95% CI: [0.03, 0.10]) and remdesivir (RR = 0.74, 95% CI: [0.62, 0.90]) were associated with lower incidence of severe adverse events. Dexamethasone was not significant in reducing disease progression (RR = 0.45, 95% CI: [0.16, 1.25]) and mortality (RR = 0.90, 95% CI: [0.70, 1.16]). The estimated combination of corticosteroids with antivirals was associated with a better clinical improvement than antivirals alone (SMD = −1.09, 95% CI: [−1.64, −0.53]).

**Conclusion:** Antivirals are safe and effective in COVID-19 treatment. Remdesivir cannot significantly reduce COVID-19 mortality and hospitalization length, while it is associated with a lower incidence of severe adverse events. Corticosteroids could increase COVID-19 severity, but it could be beneficial when combined with antivirals. Our data are potentially valuable for the clinical treatment and management of COVID-19 patients.

## Introduction

Since the outbreak of coronavirus disease 2019 (COVID-19) caused by severe acute respiratory syndrome coronavirus 2 (SARS-CoV-2) in December 2019, more than 30 million cases and 945,000 deaths have been reported as of September 17, 2020, in the world (Johns Hopkins University, 2020). Compared to other beta coronaviruses that have caused epidemics over the last 2 decades, including severe acute respiratory syndrome coronavirus (SARS-CoV) and Middle East respiratory syndrome coronavirus (MERS-CoV), SARS-CoV-2 exhibits higher infectivity while lower fatality that makes it more destructive (Abdelrahman et al., 2020a). The rapid development of effective treatment approaches for COVID-19 is urgently needed since there is no specific therapy or vaccine for COVID-19. Previous experiences from SARS and MERS treatments suggested that several interventions, including antivirals, such as lopinavir/ritonavir and umifenovir, corticosteroids, interferons, ribavirin, and newly introduced drugs, including chloroquine or its derivative hydroxychloroquine, dexamethasone, and convalescent plasma, may improve clinical outcomes in COVID-19 patients, whereas the related data are not conclusive. Since the evidence of the efficacy and safety of these treatments remains lacking, we conducted meta-analyses of the therapeutic approaches for COVID-19 patients. Our meta-analyses aimed to identify the positive benefits (such as clinical improvement) and negative risks (such as long duration of hospitalization, severe adverse events, and increased deaths) of treatment strategies for COVID-19 by comparing different treatments using different meta-analysis models. The clinical improvement of patients is referred to as the negative conversion of RT-PCR (viral loads) within 14 days or discharged alive from the hospital. On the other hand, severe side effects are referred to as shock, acute respiratory syndrome, acute kidney injury, cardiac injury, or respiratory failure.

First, we conducted a proportional meta-analysis to summarize the pooled effect of the weighted consumption proportion of each treatment. Next, we identified the relative mortality risk of patients with the treatment vs. without the treatment. Finally, we conducted the network meta-analysis to compare different treatments directly and indirectly in clinical improvement and relative mortality risk or disease progression. We summarized available randomized and non-randomized clinical trials of several treatment strategies and provided point estimates and their 95% confidence intervals (CIs) for the associations between these treatment strategies and given endpoints.

## Methods

### Search Strategy and Selection Criteria

**FIGURE 1:**
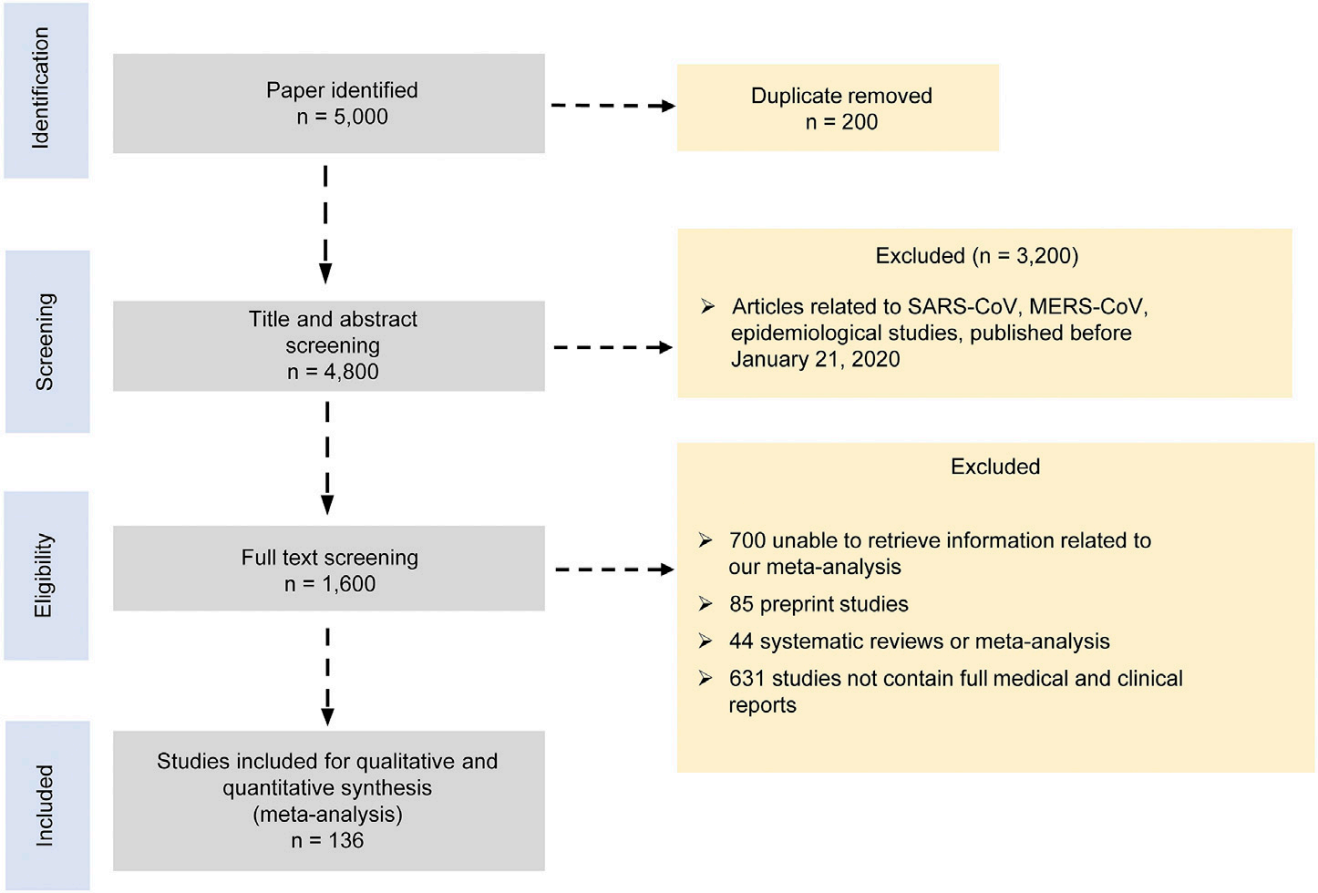
PRISMA 2009 flow diagram.

We performed this study according to the Preferred Reporting Items for Systematic reviews and Meta-Analyses recommendations “PRISMA” (PRISMA, 2015) (Figure 1 and Supplementary Table S1). We searched for publications between January 21, 2020, and September 6, 2020, in databases of PubMed, EMBASE, CNKI, Wanfang, Cochrane library, ClinicalTrials.gov, Scopus, Web of Science, Lancet, the New England Journal of Medicine, and JAMA COVID-19 platforms. We used the search term “2019 novel coronavirus, COVID-19 and SARS-CoV-2” AND “treatment, clinical characteristics, epidemiological characteristics, clinical trials, cohort studies, observational studies, case series” without language and age restriction. Search results were limited to the references published since January 21, 2020. We removed duplicate or irrelevant articles using the EndNote X9.0 software. To identify missed studies, we thoroughly checked the reference list for each selected article. Two reviewers independently screened the titles and abstracts of all references based on certain inclusion and exclusion criteria. The included studies involved the following information: clinical features, laboratory findings, and treatment approaches for COVID-19 patients with clinically defined outcomes. We excluded the following studies: 1) duplicate publications, preprints, reviews, case reports, family-based studies, unrelated titles or abstracts, studies not involving clinical features, laboratory findings, and treatment approaches, and 2) animal or *in vitro* studies. All the included studies had no restriction of age, area, and language. Two reviewers compared their screening results and discussed the differences. An agreement was reached through discussion.

### Data Extraction

Two investigators (QL and SJ) performed a literature search and data extraction, and another investigator (ZA) resolved the disagreements. We extracted the following variables: author, date, age, gender, and number of participants in different groups for comparisons, including non-survival vs. survival, treatment vs. nontreatment. The extracted data included publication date, country, study design, number of enrolled subjects, data collection method, baseline characteristics before treatment, diagnostic method, population, COVID-19 treatment details, time from admission to starting treatment, and patient outcomes.

### Data Analyses

This study performed three types of meta-analysis. First, a proportional meta-analysis by using the restricted maximum likelihood random-effect model (REML). We normalized proportional data by double-arcsine or logit transformation and confirmed their normal distribution by the Shapiro–Wilk test. Leave-one-out (LOO) analyses were performed to determine influential outliers in the proportional meta-analysis. Second, a meta-analysis of mortality risk using the Mantel–Haenszel random-effect model. Finally, a network meta-analysis evaluates direct and indirect relationships between different treatment strategies based on a common comparator, such as standard care. Standard care is defined here as supplementary oxygen, noninvasive and invasive ventilation, antibiotics, vasopressor support, RRT, and extracorporeal membrane oxygenation (Cao et al., 2020a). We estimated network meta-analysis models within a frequentist framework. We defined the clinical improvement, disease severity, and mortality as dichotomous variables and the duration of hospitalization as a continuous variable. We calculated the effect size of risk ratios (RRs) for negative outcomes and Hedges’g effect size, known as standardized mean difference (SMD), for positive outcomes with their 95% confidence intervals (CIs). The SMD results were defined as follows: small effect ≥0.2, medium effect ≥0.5, and large effect ≥0.8. We generated publication bias funnel plots by scatterplot of study effect estimates on the *x*-axis (transformed proportion or RR) against study size on the *y*-axis (standard error). The two limit lines indicate the 95% CI around the summary effect size. Egger’s test meta-regression model was used to detect funnel plot asymmetry.

Since there was clinical and methodological heterogeneity in study designs, interventions, and outcome measures, and participants’ characteristics, a random-effect model was adopted in meta-analyses. Additionally, the Sidik–Jonkman estimator, tau^^2^, and the confidence interval of tau^2^ were used. The Hartung–Knapp method was used to adjust for the random-effect model. *I* ^2^ statistic and Cochran’s Q test were used to assess statistical heterogeneity/inconsistency among meta-analysis models with a threshold of I^2^ > 50% or *p* ≤ 0.05. We performed meta-analyses using R packages “meta, netmeta, dmetar, and metafor” (Wang, 2018).

### Study Selection and Risk of Bias Assessment

We assessed the risk of bias for eligible observational studies, such as cross-sectional, cohort studies, and case series, following the Strengthening the Reporting of Observational Studies in Epidemiology (STROBE) reporting guidelines (von Elm et al., 2007). We assessed the risk of bias in randomized control trials using the Cochrane risk-of-bias tool for randomized trials (ROB-2) (Higgins et al., 2011). Two investigators (QL and SJ) conducted both risks of bias evaluations independently. Each assigned an overall risk of bias to each eligible study and consulted a third reviewer (ZA) if they disagreed. We summarized the results in Supplementary Figure S1.

## Results

We initially identified 5,000 articles based on our search criteria, most of which were irrelevant to our research objective or without published results (Figure 1). Finally, we included 136 articles in our meta-analysis, which involved a total of 102,345 subjects (Alam et al., 2020; Almazeedi et al., 2020; Asai et al., 2020; Bakhshaliyev et al., 2020; Beigel et al., 2020; Berenguer et al., 2020; Bhandari et al., 2020; Bhatraju et al., 2020; Boulware et al., 2020; Buckner et al., 2020; Cai et al., 2020; Campochiaro et al., 2020; Cao et al., 2020a; Cao et al., 2020b; Capone et al., 2020; Casas-Rojo et al., 2020; Catteau et al., 2020; Chen et al., 2020a; Chen et al., 2020b; Chen et al., 2020c; Chen et al., 2020d; Chen Jun et al., 2020; Colaneri et al., 2020; Deng et al., 2020; Dequin et al., 2020; Di Lorenzo et al., 2020; Fauvel et al., 2020; Ferrando et al., 2020; Fried et al., 2020; García−Salido et al., 2020; Gautret et al., 2020; Geleris et al., 2020; Grein et al., 2020; Guervilly et al., 2020; Gupta et al., 2020; Han et al., 2020; Heffler et al., 2020; Hong et al., 2020; Hu et al., 2020a; Hu et al., 2020b; Huang et al., 2020a; Huang et al., 2020b; Huang et al., 2020c; Hung et al., 2020; Hur et al., 2020; Jacobs et al., 2020; Jiang et al., 2020; Karolyi et al., 2020; Kaushik et al., 2020; Khamis et al., 2020; Kim et al., 2020a; Kim et al., 2020b; Koleilat et al., 2020; Krishnan et al., 2020; Lecronier et al., 2020; Lee et al., 2020a; Lee et al., 2020b; Lendorf et al., 2020; Li et al., 2020a; Li et al., 2020b; Liabeuf et al., 2020; Lian et al., 2020a; Lian et al., 2020b; Liu et al., 2020a; Liu et al., 2020b; Liu et al., 2020c; Liu et al., 2020d; Lo et al., 2020; Lokken et al., 2020; Lovato et al., 2020; Maeda et al., 2020; Mahmoudi et al., 2020; Mikami et al., 2020; Mo et al., 2020; Monfared et al., 2020; Montastruc et al., 2020; Montero et al., 2020; Morena et al., 2020; Moreno−Pérez et al., 2020; Morrison et al., 2020; Mostaza et al., 2020; Myers et al., 2020; Nguyen et al., 2020; Nowak et al., 2020; Okoh et al., 2020; Ouyang et al., 2020; Palmieri et al., 2020; Pan et al., 2020; Pasquini et al., 2020; Pelayo et al., 2020; Piva et al., 2020; Potere et al., 2020; Price et al., 2020; Primmaz et al., 2020; Quartuccio et al., 2020; Richardson et al., 2020; Recovery Collaborative Group, 2020; Rosenberg et al., 2020; Salacup et al., 2020; Saleh et al., 2020; Sentilhes et al., 2020; Shah et al., 2020; Shekerdemian et al., 2020; Shi et al., 2020; Singh et al., 2020b; Skipper et al., 2020; Spinner et al., 2020; Suleyman et al., 2020; Tang et al., 2020; The Writing Committee for the REMAP-CAP Investigators, 2020; Tomazini et al., 2020; Toniati et al., 2020; Vahidy et al., 2020; Wang et al., 2020a; Wang et al., 2020b; Wang et al., 2020d; Wang et al., 2020e; Wu et al., 2020a; Wu et al., 2020b; Wu et al., 2020c; Xu et al., 2020a; Xu et al., 2020b; Xu et al., 2020c; Yan et al., 2020a; Yan et al., 2020b; Yang et al., 2020; Yu et al., 2020a; Yu et al., 2020b; Zerah et al., 2020; Zhang et al., 2020a; Zhang et al., 2020b; Zhang et al., 2020c; Zhao et al., 2020; Zheng et al., 2020; Zhou et al., 2020; Zhu et al., 2020).

### Characteristics of the Included Studies

The 136 studies, which involved 102,345 patients, included 97 observational studies, 11 case series, five letters, one application note, 13 randomized trials, one pilot study, five reports, one non-randomized trial, one short communication, and one correspondence. The countries involved in these studies included China (51 studies (Cai et al., 2020; Cao et al., 2020a; Cao et al., 2020b; Chen et al., 2020a; Chen et al., 2020b; Chen et al., 2020c; Chen et al., 2020d; Chen Jun et al., 2020; Deng et al., 2020; Hu et al., 2020a; Hu et al., 2020b; Huang et al., 2020a; Huang et al., 2020b; Huang et al., 2020c; Hung et al., 2020; Jiang et al., 2020; Li et al., 2020a; Li et al., 2020b; Lian et al., 2020a; Lian et al., 2020b; Liu et al., 2020a; Liu et al., 2020b; Liu et al., 2020c; Liu et al., 2020d; Mo et al., 2020; Ouyang et al., 2020; Pan et al., 2020; Shi et al., 2020; Tang et al., 2020; Wang et al., 2020a; Wang et al., 2020b; Wang et al., 2020d; Wang et al., 2020e; Wu et al., 2020a; Wu et al., 2020b; Wu et al., 2020c; Xu et al., 2020a; Xu et al., 2020b; Xu et al., 2020c; Yan et al., 2020a; Yan et al., 2020b; Yang et al., 2020; Yu et al., 2020a; Yu et al., 2020b; Zhang et al., 2020a; Zhang et al., 2020b; Zhang et al., 2020c; Zhao et al., 2020; Zheng et al., 2020; Zhou et al., 2020; Zhu et al., 2020)), the United States (31 studies (Alam et al., 2020; Bhatraju et al., 2020; Buckner et al., 2020; Capone et al., 2020; Fried et al., 2020; Geleris et al., 2020; Grein et al., 2020; Gupta et al., 2020; Hur et al., 2020; Jacobs et al., 2020; Kaushik et al., 2020; Kim et al., 2020a; Koleilat et al., 2020; Krishnan et al., 2020; Lokken et al., 2020; Maeda et al., 2020; Mikami et al., 2020; Morrison et al., 2020; Myers et al., 2020; Okoh et al., 2020; Pelayo et al., 2020; Price et al., 2020; Richardson et al., 2020; Rosenberg et al., 2020; Salacup et al., 2020; Saleh et al., 2020; Shah et al., 2020; Shekerdemian et al., 2020; Singh et al., 2020b; Suleyman et al., 2020; Vahidy et al., 2020)), France (10 studies (Dequin et al., 2020; Fauvel et al., 2020; Gautret et al., 2020; Guervilly et al., 2020; Lecronier et al., 2020; Liabeuf et al., 2020; Montastruc et al., 2020; Nguyen et al., 2020; Sentilhes et al., 2020; Zerah et al., 2020)), Italy (12 studies (Campochiaro et al., 2020; Colaneri et al., 2020; Di Lorenzo et al., 2020; Heffler et al., 2020; Lovato et al., 2020; Morena et al., 2020; Palmieri et al., 2020; Pasquini et al., 2020; Piva et al., 2020; Potere et al., 2020; Quartuccio et al., 2020; Toniati et al., 2020)), Korea (four studies (Han et al., 2020; Hong et al., 2020; Kim et al., 2020b; Lee et al., 2020a)), Kuwait (one study (Almazeedi et al., 2020)), Japan (one study (Asai et al., 2020)), Turkey (one study (Bakhshaliyev et al., 2020)), Spain (seven studies (Berenguer et al., 2020; Casas-Rojo et al., 2020; Ferrando et al., 2020; García-Salido et al., 2020; Montero et al., 2020; Moreno-Pérez et al., 2020; Mostaza et al., 2020)), India (one study (Bhandari et al., 2020)), Canada (one study (Boulware et al., 2020)), Belgium (one study (Catteau et al., 2020)), Oman (one study (Khamis et al., 2020)), Singapore (one study (Lee et al., 2020b)), Denmark (one study (Lendorf et al., 2020)), Iran (two studies (Mahmoudi et al., 2020; Monfared et al., 2020)), United Kingdom (one study (Recovery Collaborative Group, 2020)), Switzerland (two studies (Pellaud et al., 2020; Primmaz et al., 2020)), Poland (one study (Nowak et al., 2020)), Brazil (one study (Tomazini et al., 2020)), Macau (one study (Lo et al., 2020)), Austria (one study (Karolyi et al., 2020)), and three studies (Beigel et al., 2020; Spinner et al., 2020; The Writing Committee for the REMAP-CAP Investigators, 2020) from multiple countries. A detailed description of these studies is shown in Supplementary Table S2.

Of the 136 studies, 45 reported antibiotics as one of the main treatment strategies for COVID-19, 58 for antivirals, 43 for corticosteroids, 27 for chloroquine or hydroxychloroquine, 19 for immunoglobulins, nine for interferons, 15 for RRT, 14 for tocilizumab, and less than five for IL-6 antagonist, immunomodulators, or immune enhancers. This study focused on evaluating the frequently used treatment strategies, including antibiotics, antivirals, corticosteroids, chloroquine or hydroxychloroquine, tocilizumab, interferons, immunoglobulins, and RRT.

### Proportional Meta-analysis

**FIGURE 2:**
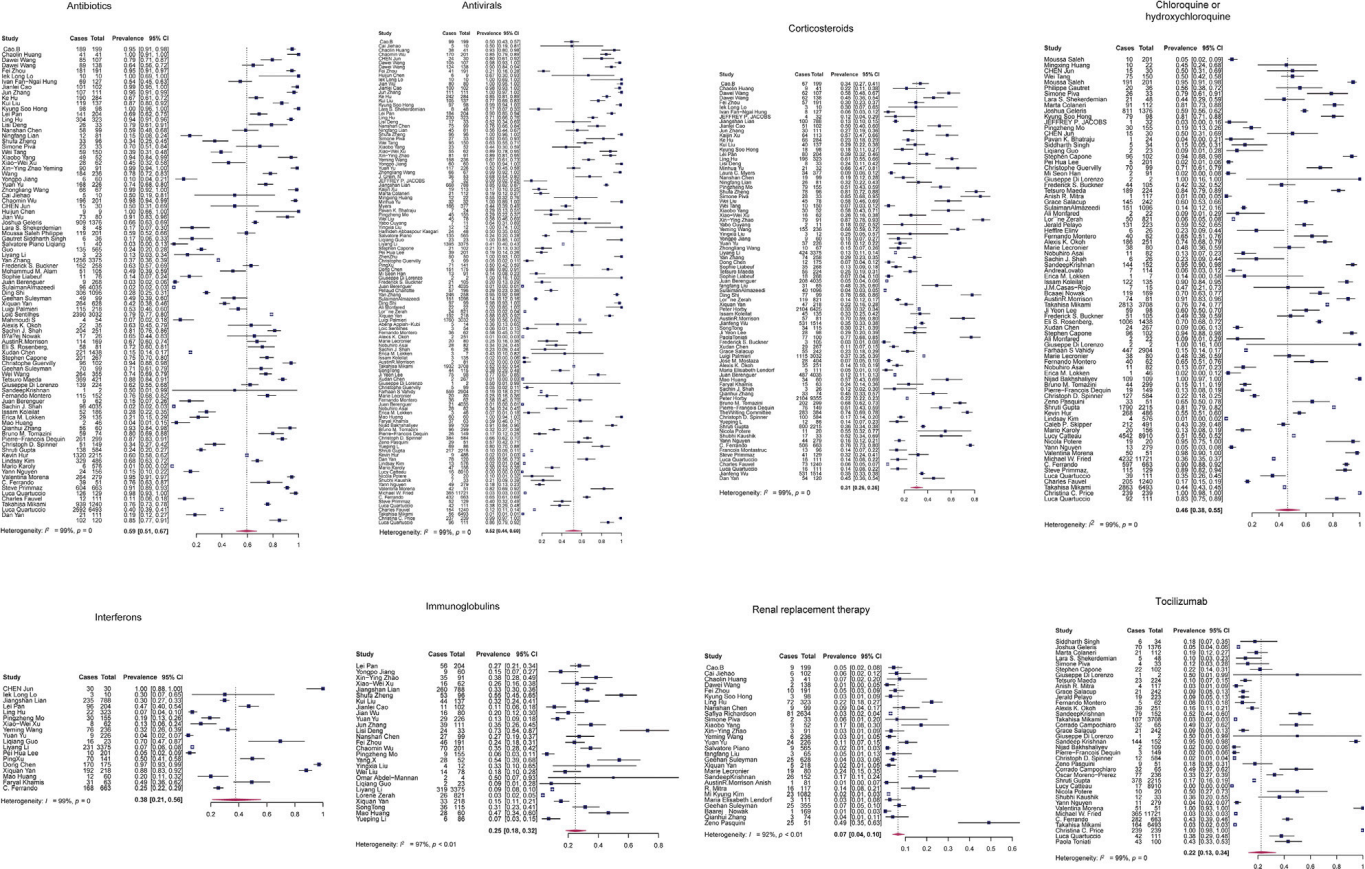
Proportional meta-analysis forest plots of the eight treatment strategies for COVID-19 by the random-effect model. Cases, number of patients taking the drug; total, the total number of patients enrolled in the study.

First, we performed a proportional meta-analysis to estimate binomial (weighted) consumption proportions of each treatment. We obtained the average of the proportions of multiple studies weighted by the inverse of their sampling variances using the random-effect model. The sample sizes in these studies ranged from 7 to 15,111, with a total number of 102,345. The high prevalence was observed in antibiotics (proportion: 0.59, 95% CI: [0.51, 0.67]) and antivirals (proportion: 0.52, 95% CI: [0.44, 0.60]) (Figure 2). We showed the results of proportional meta-analysis heterogeneity in Supplementary Table S3.

To determine whether outliers were influencing the proportional analysis results, we performed leave-one-out (LOO) analyses. We found six of the eight models having significant outliers, including antibiotics, antivirals, chloroquine or hydroxychloroquine, corticosteroids, interferons, and tocilizumab. Immunoglobulins and RRT models did not show any significant outliers (Supplementary Figures S2, S3). Finally, we assessed the potential publication bias in each treatment model using a funnel plot of the mixed-effect meta-regression model and the Egger’s regression test with the standard error as a predictor. The funnel plots were roughly symmetrical in antibiotics, antivirals, chloroquine or hydroxychloroquine, interferons, and tocilizumab (*p* > 0.05). In contrast, Egger’s test was significant in three treatment models: corticosteroids, immunoglobulins, and RRT (*p* < 0.05; Supplementary Figure S4).

**FIGURE 3:**
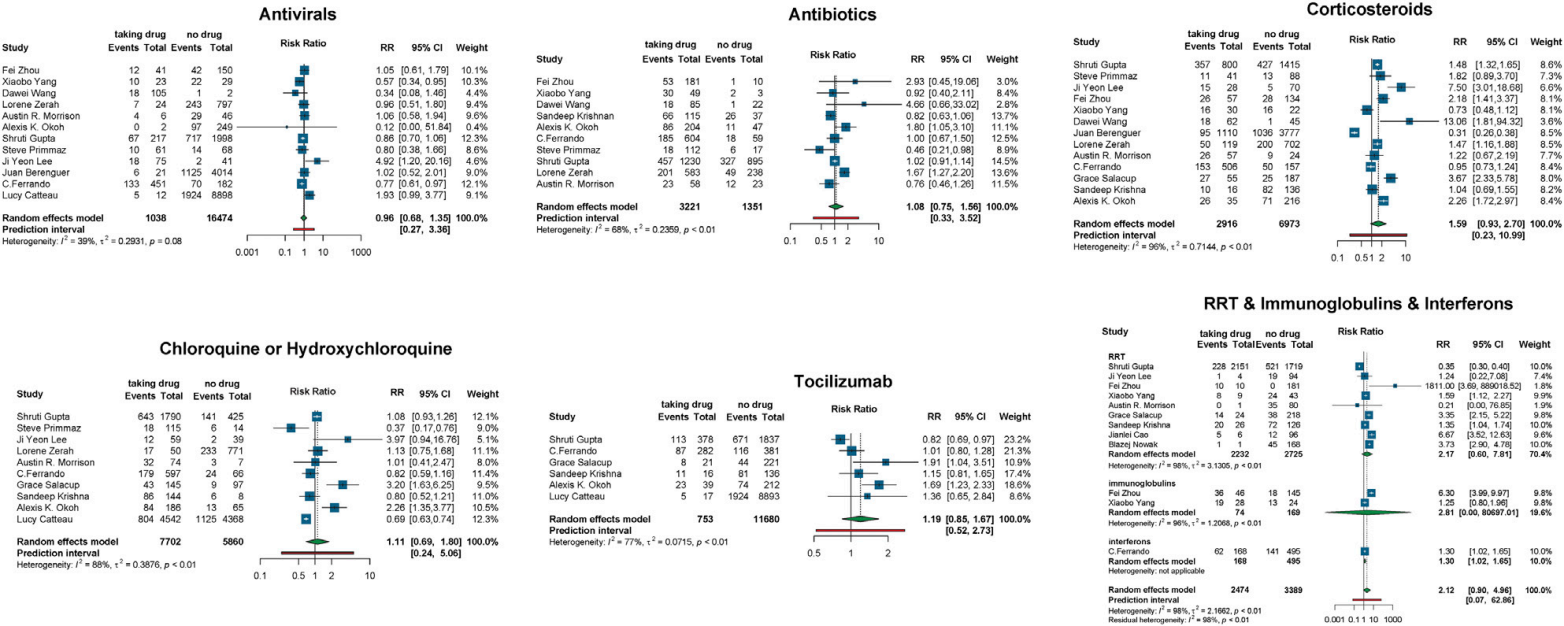
Relative mortality risk in COVID-19 patients receiving vs. not receiving therapy. Events, number of patients died from COVID-19; total, the total number of patients taking/not taking the drug; RR, risk ratio; CI, confidence interval.

Second, to estimate the association between each treatment and COVID-19 mortality, we performed a meta-analysis of mortality risk using the Mantel–Haenszel random-effect model to quantify each drug effect on COVID-19 mortality. We used the general term “relative risk” to refer to the mortality risk. A total of 16 observational studies (Berenguer et al., 2020; Cao et al., 2020b; Catteau et al., 2020; Ferrando et al., 2020; Gupta et al., 2020; Krishnan et al., 2020; Lee et al., 2020a; Morrison et al., 2020; Nowak et al., 2020; Okoh et al., 2020; Primmaz et al., 2020; Salacup et al., 2020; Wang et al., 2020a; Yan et al., 2020b; Zerah et al., 2020; Zhou et al., 2020) were involved in this assessment. We extracted the number of patients who died from COVID-19 following a certain treatment. We found that the use of interferons increases the risk of death (RR = 1.30, 95% CI: [1.02, 1.65]). In contrast, antibiotics, antivirals, chloroquine or hydroxychloroquine, corticosteroids, tocilizumab, RRT, and immunoglobulins did not show any significant impact on COVID-19 patients’ mortality (Figure 3).

### Network Meta-Analysis

**FIGURE 4:**
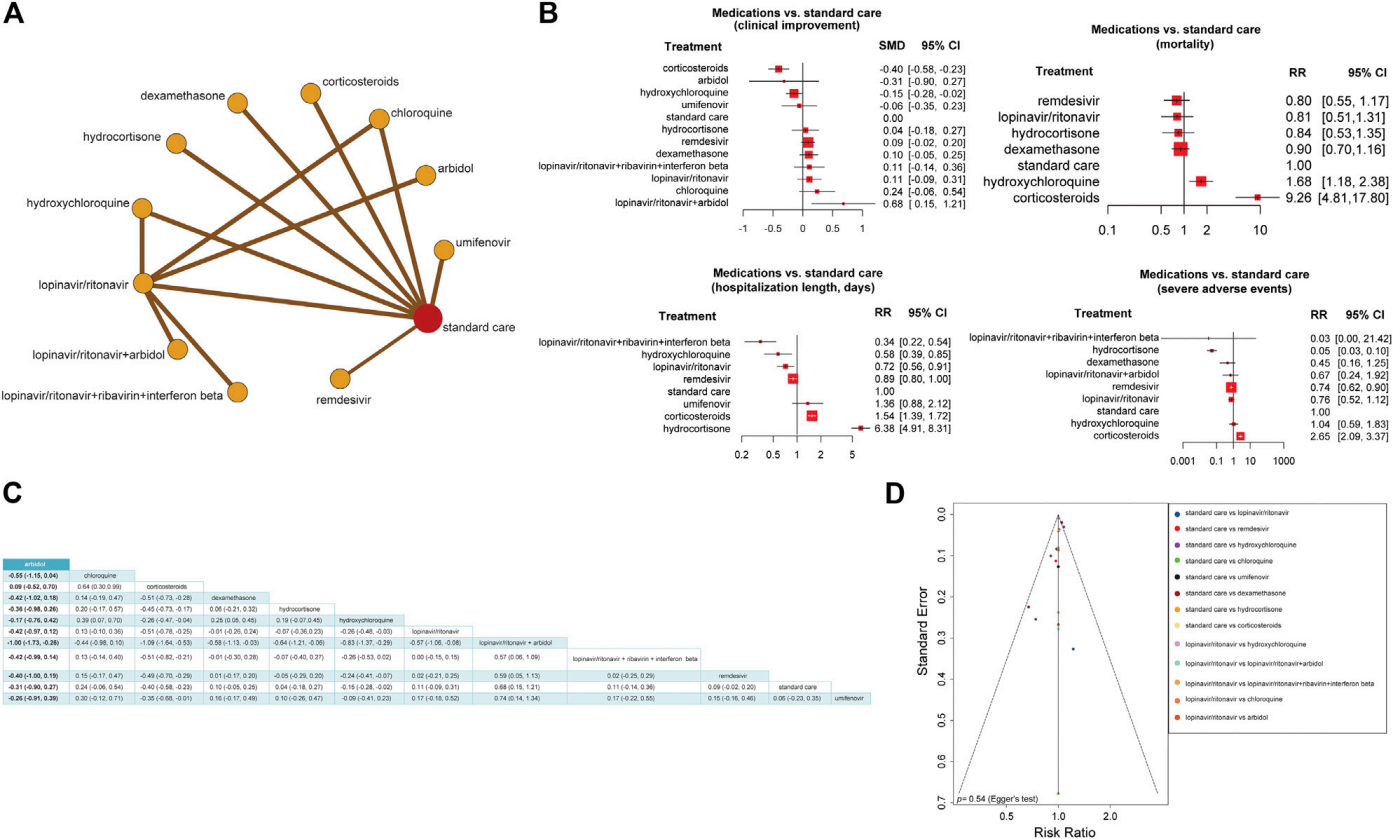
Network meta-analysis to compare the effect of different anti–COVID-19 treatment approaches. **(A)** Network frequentist model of 12 treatment approaches for COVID-19. The nodes represent treatments, and the edges represent comparisons of treatments indicated in studies. These results were obtained by the frequentist random-effect model. **(B)** Forest plots of treatment vs. standard care based on four outcomes: clinical improvement, mortality, length of hospitalization (days), and severe side effects. RR, risk ratio; SMD, standardized mean difference; CI, confidence interval. **(C)** The matrix for the effect sizes of all possible treatment combinations. The standardized mean difference and their 95% confidence intervals are shown generated by the random-effect model. **(D)** Comparison-adjusted funnel plot showing the network meta-analysis publication bias by testing the funnel plot asymmetry using Egger’s regression test.

For further validation, we compare the efficacy and safety of different anti–COVID-19 treatments by a network meta-analysis, which included 20 studies (Beigel et al., 2020; Cao et al., 2020a; Deng et al., 2020; Dequin et al., 2020; Gautret et al., 2020; Geleris et al., 2020; Huang et al., 2020b; Hung et al., 2020; Karolyi et al., 2020; Lecronier et al., 2020; Lian et al., 2020b; Recovery Collaborative Group, 2020; Skipper et al., 2020; Spinner et al., 2020; Tang et al., 2020; The Writing Committee for the REMAP-CAP Investigators, 2020; Tomazini et al., 2020; Wang et al., 2020d; Wu et al., 2020b; Zhu et al., 2020) (Supplementary Table S4). These studies had identified treatment groups as drug vs. standard care or standard treatments. First, we used the frequentist network model to define the effect size of each comparison as RR and the network inconsistency as the Q value. We did not observe any network inconsistency among our model (0.46, 95% CI: [0.00, 0.75]); heterogeneity within the design: Q = 9.21, *p* = 0.16; heterogeneity between designs: Q = 5.62, *p* = 0.06). Next, we estimated the efficacy and safety of each treatment relative to the standard care (Figure 4A). We found that the combination of lopinavir/ritonavir and Arbidol was significantly effective among COVID-19 treatments (SMD = 0.68, 95% CI: [0.15, 1.21]) compared to standard care. Arbidol, chloroquine or hydroxychloroquine, umifenovir, hydrocortisone, remdesivir, lopinavir/ritonavir, and the combination of lopinavir/ritonavir with ribavirin and interferon-β showed no significant impact on clinical improvement compared to standard care (Figure 4B).

The use of corticosteroids was associated with a higher risk of disease progression and death (mortality: RR = 9.26, 95% CI: [4.81, 17.80]; hospitalization length: RR = 1.54, 95% CI: [1.39, 1.72]; severe side effect: RR = 2.65, 95% CI: [2.09, 3.37]), although it showed a small clinical improvement in COVID-19 patients (SMD = −0.40, 95% CI: [−0.85, −0.23]). The use of hydroxychloroquine was also associated with a higher risk of death (RR = 1.68, 95% CI: [1.18, 2.38]) than standard care. Remdesivir, lopinavir/ritonavir, and hydrocortisone showed no significant impact on reducing mortality risk compared to standard care (Figure 4B).

The combination of lopinavir/ritonavir, ribavirin, and interferon-β (RR = 0.34, 95% CI: [0.22, 0.54]), hydroxychloroquine (RR = 0.58, 95% CI: [0.39, 0.58]), and lopinavir/ritonavir (RR = 0.72, 95% CI: [0.56, 0.91]) was associated with reduced hospitalization length compared to standard care. The use of hydrocortisone (RR = 6.38, 95% CI: [4.91, 8.31]) was associated with prolonged hospitalization length compared to standard care. Remdesivir and umifenovir showed no significant impact on reducing hospitalization length compared to standard care (Figure 4B).

The use of hydrocortisone (RR = 0.05, 95% CI: [0.03, 0.10]) and remdesivir (RR = 0.74, 95% CI: [0.62, 0.90]) was associated with lower incidence of severe adverse events than standard care. The combination of lopinavir/ritonavir with ribavirin and interferon-β, besides the combination of lopinavir/ritonavir with Arbidol and hydroxychloroquine, showed no significant association with a lower or higher incidence of severe adverse events compared to standard care (Figure 4B).

Dexamethasone, a newly FDA approved corticosteroid for treating COVID-19, did not show any significant association with lower or higher incidence of severe adverse events (RR = 0.45, 95% CI: [0.16, 1.25]), mortality (RR = 0.90, 95% CI: [0.70, 1.16]), or clinical improvement (SMD = −0.10, 95% CI: [−0.05, 0.25]), compared to standard care (Figure 4B). Furthermore, we produced a matrix for the effect sizes of all possible treatment combinations. We estimated that the combination of lopinavir/ritonavir and Arbidol with corticosteroids may be effective (SMD = −1.09, 95% CI: [−1.64, −0.53]). Besides, the combination of lopinavir/ritonavir and Arbidol (SMD = −1.00, 95% CI: [−1.73, −0.26]) and lopinavir/ritonavir and dexamethasone (SMD = −0.58, 95% CI: [−1.13, −0.03]) may have resulted in a good clinical improvement (Figure 4C).

Since assessing the publication bias of a network meta-analysis in its aggregated form is problematic (Asai et al., 2020), we conducted an analysis called “comparison-adjusted funnel plot.” We did not observe the funnel asymmetry (Egger’s Test, *p* = 0.54; Figure 4D).

## Discussion and Conclusion

Currently, most of the COVID-19 treatments are symptomatic treatments. Treatments and oxygen therapy are the primary steps in addressing respiratory impairment in COVID-19 patients. Accumulating knowledge on the pathophysiology of lung damage provides clinicians with the strategies for dealing with respiratory failure caused by COVID-19 (Abdelrahman et al., 2020b). Several treatment attempts have become an essential part of COVID-19 treatment and management protocol, including antibiotics, antivirals, corticosteroids, interferons, RRT, chloroquine or hydroxychloroquine, dexamethasone, and tocilizumab, whereas there were no clear recommendations or rationale for using them. Earlier, 58% of COVID-19 patients in Wuhan were treated with antibiotics, suspecting that lung inflammation is mostly correlated with bacterial infections. Later, the WHO recommended using empiric antibiotics against the bacterial superinfections of COVID-19 patients (Ginsburg and Klugman, 2020), although we did not find any significant impact of antibiotics on reducing mortality rates of COVID-19 patients.

During the SARS outbreak in 2004, serum concentrations of IL-6, IL-8, IL-16, and tumor necrosis factor-α were markedly upregulated in SARS patients (Gomersall, 2004). Since the median duration of ICU stays in SARS and COVID-19 patients ranged from 8 to 15 days, a logical scenario was introduced to modify the immune response with anti-inflammatory agents, such as corticosteroids (Gomersall, 2004). Although the data on the patients with acute respiratory distress syndrome (ARDS) showed that high-dose corticosteroids might have a beneficial effect, especially in mitigating the destructive inflammatory response in severe COVID-19 patients (Wu et al., 2020b), the rationale for using corticosteroids is still controversial due to their high adverse risk profile (Singh et al., 2020a). Recently, a few studies declared that dexamethasone could result in a lower risk of mortality than other corticosteroids or standard care (Recovery Collaborative Group, 2020; Tomazini et al., 2020). We found that corticosteroids were associated with a higher risk of disease progression and death than standard care and that dexamethasone neither significantly reduces severe side effects or mortality nor enhances clinical improvements compared to other corticosteroids or standard care.

Additionally, our results indicate that the use of corticosteroids has a positive impact on outcomes in COVID-19 patients compared to standard care, but the impact is minor. Furthermore, some reports stated that using corticosteroids in small doses could benefit COVID-19 patients by mitigating inflammatory response (Wu et al., 2020b), consistent with the results from our network analysis. However, the risk of using corticosteroids often outweighs its benefits (Wu et al., 2020b), especially in high doses, although the FDA has approved dexamethasone for COVID-19 treatment. Our results showed that dexamethasone had no significantly higher efficacy or safety than other drugs compared to standard care. Still, the combination of dexamethasone and antivirals could have more benefits for COVID-19 patients than dexamethasone alone, as suggested by our indirect treatment combination analysis (Figure 4C). Thus, corticosteroids could be beneficial in COVID-19 treatment if they combine in small doses with other drugs, although further clinical trials are needed to prove this.

The effectiveness of chloroquine or hydroxychloroquine against SARS-CoV-2 has been proposed in several studies (Bakhshaliyev et al., 2020; Boulware et al., 2020). Chloroquine and hydroxychloroquine are antimalarial drugs, which are also used for treating autoimmune diseases, such as systemic lupus erythematosus and rheumatoid arthritis (Bakhshaliyev et al., 2020; Boulware et al., 2020). *In vitro* studies showed that chloroquine and hydroxychloroquine could inhibit the fusion of SARS-CoV-2 with the host cell membranes by increasing the endosomal pH and interfere with the binding of SARS-CoV-2 to the cell receptor angiotensin-converting enzyme-2 (ACE2) by inhibiting its glycosylation (Sarayani et al., 2020). Although these studies have provided potential mechanisms of chloroquine and hydroxychloroquine in the treatment of COVID-19, the clinical evidence for their effectiveness remains lacking. Our results demonstrated that the use of hydroxychloroquine was associated with higher COVID-19 mortality risk, while it significantly reduced hospitalization length of COVID-19 patients compared to the standard care. This positive impact might be based on the prescribed dose or the patient’s clinical status. However, one study in Belgium was conducted to evaluate the efficacy of low-dose hydroxychloroquine on COVID-19 mortality. They determined that a low-dose hydroxychloroquine was independently associated with lower mortality in hospitalized COVID-19 patients than standard care alone (Catteau et al., 2020). Another clinical trial was conducted on whether using hydroxychloroquine alone or with azithromycin could improve the clinical status of mild/moderate COVID-19 patients within 15 days or not. The results revealed that there is no significant improvement in these patients within 15 days (Cavalcanti et al., 2020). Although the World Health Organization (WHO) recommends against using these drugs due to the limited evidence, most African countries are still utilizing them as the routine COVID-19 treatment (Belayneh, 2020).

Arbidol, a drug targeting influenza hemagglutinin (HA), was used in an early clinical trial of COVID-19 treatment (ChiCTR2000029573) and was consequently recommended in the Guidelines for the Diagnosis and Treatment of COVID-19 (sixth and seventh editions) in China (Wang et al., 2020c). Several studies have suggested that Arbidol could improve hospital discharge rate and reduce the risk of COVID-19 mortality (Li et al., 2020b; Deng et al., 2020). Likewise, lopinavir/ritonavir showed a broad safety profile, especially in mitigating disease progression and severity (Karolyi et al., 2020). Our study showed that the combination of lopinavir/ritonavir and Arbidol was the most effective and safest among all COVID-19 treatments relative to the standard care. Also, the use of lopinavir/ritonavir significantly reduced hospitalization length compared to standard care. Remdesivir has been recognized as a promising antiviral drug against various RNA viruses, including SARS-CoV and MERS-CoV (Sheahan et al., 2017). However, its adverse effects have become a concern with its increased application in COVID-19 patients. Several studies have reported that remdesivir might increase liver injury (The COVID-19 Investigation Team, 2020), gastrointestinal discomfort (The COVID-19 Investigation Team, 2020), respiratory toxicity (Spinner et al., 2020), cardiovascular toxicity, and nephrotoxicity (Grein et al., 2020; Spinner et al., 2020). Recently, the WHO has issued a conditional recommendation against the use of remdesivir in hospitalized patients regardless of their disease severity, considering that there is no evidence that remdesivir can improve survival prognosis and other outcomes in COVID-19 patients (WHO, 2020). Our result demonstrated that using remdesivir was associated with a lower incidence of severe adverse events, whereas further clinical trials are required for validation of this result.

Since the outbreak of COVID-19, the guidelines and recommendations for COVID-19 treatment have been altered several times by the Centers for Disease Control and Prevention (CDC) and the National Institutes of Health (NIH). Since COVID-19 is often accompanied by organ damage caused by an inflated immune or inflammatory response to SARS-CoV-2 infection, the recommendations suggest that antiviral therapies would have the most significant effect for the early stage of COVID-19. For the late stage of COVID-19, immunosuppressive/anti-inflammatory treatments would be more effective. Nevertheless, currently, there are inadequate data from clinical trials for or against the use of a specific therapy. Hence, the COVID-19 Treatment Guidelines Panel keeps updating treatment recommendations for COVID-19 by reviewing the most recent clinical data (Nih Coronavirus Disease, 2019).

The study has several limitations. First, we may not include some relevant studies due to certain restrictions in searching for databases. Second, the quality of evidence is limited by data primarily derived from retrospective analyses, including heterogeneous data and study designs. Third, we could not conduct subgroup analyses due to the limited number of studies. Fourth, in some situations, we reported wide CIs because of clinical and methodological heterogeneity in studies, even when random-effect models were adopted. Finally, the number of randomized clinical trials included in network meta-analysis is limited, and thus further randomized trials are essential for validation and interpretation of the results.

In conclusion, this is the first systematic review, meta-analysis, and network meta-analysis of the efficacy and safety of eight COVID-19 therapeutic approaches. We believe that our study is potentially valuable for the clinical treatment and management of COVID-19 patients. This study performed three types of meta-analysis, namely, proportional meta-analysis to account for the average proportion of each treatment, mortality risk meta-analysis to estimate treatment risk on COVID-19 mortality, and network meta-analysis to compare the efficacy and safety of different COVID-19 treatments. Our results favor the routine use of antivirals against COVID-19 because of their wide safety and efficacy profiles, especially lopinavir/ritonavir without associations with disease progression and mortality. Our results did not support the routine use of remdesivir due to its uncertain safety and efficacy profiles. In addition, our results suggest that corticosteroids could increase COVID-19 severity, but it could be beneficial when combining with antivirals in small doses.

This systematic review and network meta-analysis would form part of a living or evolving meta-analysis or network meta-analysis and is required to be periodically updated as further new randomized trials that examined remdesivir, glucocorticoids, lopinavir/ritonavir, and chloroquine or hydroxychloroquine will be available soon.

## Data Availability

The original contributions presented in the study are included in the article/Supplementary Material; further inquiries can be directed to the corresponding author.
