# Supplementary material for: Evaluation of the Current Therapeutic Approaches for COVID-19: A Systematic Review and a Meta-analysis
Source: Front Pharmacol. 2021 Mar 15;12:607408. doi: 10.3389/fphar.2021.607408 (PMC8005525; doi:10.3389/fphar.2021.607408)
Supplement: Supplementary file 1 [file datasheet1.pdf]

Supplementary Materials

Supplementary Table 1. PRISMA 2009 Checklist.

Supplementary Table 2. Characteristics of the 136 studies included in the meta-analysis.

Supplementary Table 3. Heterogeneity results from the proportional meta-analysis.

Supplementary Table 4. A summary of the 20 studies included in the network meta-analysis.

Supplementary Figure 1. Cochrane Risk of bias analysis (ROB-2) of five randomized trial studies of COVID-19 treatment.

Supplementary Figure 2. Subgroup meta-analysis of eight treatments to estimate the impact of each treatment on the severity of COVID-19. The forest plots showing the treatment effect on different subgroups (severe versus non-severe, ICU versus non-ICU, non-severe versus survival, and deterioration versus discharge) using the Mantel-Haenszel random-effect model.

Supplementary Figure 3. Diagnostic plots showing significant and influential studies by analyses of externally studentized residuals, difference in fits values, Cook's distances, covariance ratios, tau2 ,and Q-test of leave-one-out heterogeneity, hat values, and weights.

Supplementary Figure 4. Funnel plots showing proportional meta-analysis publication bias by testing for the funnel plot asymmetry using the Egger's regression test. The Z and P values are shown.

Supplementary Table 1. PRISMA 2009 Checklist

Section/topic	#	Checklist item	Reported on page #
TITLE			
Title	1	Identify the report as a systematic review, meta-analysis, or both.	1
ABSTRACT			
Structured summary	2	Provide a structured summary including, as applicable: background, objectives, data sources, study eligibility criteria, participants, and interventions, study appraisal and synthesis methods, results, limitations, conclusions and implications of key findings, systematic review registration number.	2-3
INTRODUCTION			
Rationale	3	Describe the rationale for the review in the context of what is already known.	3
Objectives	4	Provide an explicit statement of questions being addressed with reference to participants, interventions, comparisons, outcomes, and study design (PICOS).	3
METHODS			
Protocol and registration	5	Indicate if a review protocol exists, if and where it can be accessed (e.g., Web address), and, if available, provide registration information including registration number.	-
Eligibility criteria	6	Specify study characteristics (e.g., PICOS, length of follow-up) and report characteristics (e.g., years considered, language, publication status) used as criteria for eligibility, giving rationale.	3-5
Information sources	7	Describe all information sources (e.g., databases with dates of coverage, contact with study authors to identify additional studies) in the search and date last searched.	3-5
Search	8	Present full electronic search strategy for at least one database, including any limits used, such that it could be repeated.	4
Study selection	9	State the process for selecting studies (i.e., screening, eligibility, included in systematic review, and, if applicable, included in the meta-analysis).	4
Data collection process	10	Describe method of data extraction from reports (e.g., piloted forms, independently, in duplicate) and any processes for obtaining and confirming data from investigators.	3-5
Data items	11	List and define all variables for which data were sought (e.g., PICOS, funding sources) and any assumptions and simplifications made.	3-5

Section/topic	#	Checklist item	Reported on page #
Risk of bias in individual studies	12	Describe methods used for assessing risk of bias of individual studies (including specification of whether this was done at the study or outcome level), and how this information is to be used in any data synthesis.	5
Summary measures	13	State the principal summary measures (e.g., risk ratio, difference in means).	4-5
Synthesis of results	14	Describe the methods of handling data and combining results of studies, if done, including measures of consistency (e.g., I^2) for each meta-analysis.	4-5
Risk of bias across studies	15	Specify any assessment of risk of bias that may affect the cumulative evidence (e.g., publication bias, selective reporting within studies).	5
Additional analyses	16	Describe methods of additional analyses (e.g., sensitivity or subgroup analyses, meta-regression), if done, indicating which were pre-specified.	4-5
RESULTS			
Study selection	17	Give numbers of studies screened, assessed for eligibility, and included in the review, with reasons for exclusions at each stage, ideally with a flow diagram.	5
Study characteristics	18	For each study, present characteristics for which data were extracted (e.g., study size, PICOS, follow-up period) and provide the citations.	5
Risk of bias within studies	19	Present data on risk of bias of each study and, if available, any outcome level assessment (see item 12).	6-7
Results of individual studies	20	For all outcomes considered (benefits or harms), present, for each study: (a) simple summary data for each intervention group (b) effect estimates and confidence intervals, ideally with a forest plot.	6-7
Synthesis of results	21	Present results of each meta-analysis done, including confidence intervals and measures of consistency.	6-7
Risk of bias across studies	22	Present results of any assessment of risk of bias across studies (see Item 15).	5
Additional analysis	23	Give results of additional analyses, if done (e.g., sensitivity or subgroup analyses, meta-regression [see Item 16]).	-
DISCUSSION			
Summary of evidence	24	Summarize the main findings including the strength of evidence for each main outcome, consider their relevance to key groups (e.g., healthcare providers, users, and policy makers).	7-9
Limitations	25	Discuss limitations at study and outcome level (e.g., risk of bias), and at review-level (e.g., incomplete retrieval of identified research, reporting bias).	9

Section/topic	#	Checklist item	Reported on page #
Conclusions	26	Provide a general interpretation of the results in the context of other evidence, and implications for future research.	8-9
FUNDING			
Funding	27	Describe sources of funding for the systematic review and other support (e.g., supply of data), role of funders for the systematic review.	9

Supplementary Table *2*. Characteristics of the 136 studies included in the meta-analysis.

Treatment	Study design	Origin	Number of cases	Study
antivirals, corticosteroids	retrospective	China	32	Minhua Yu et al. [1]
antivirals, corticosteroids	retrospective	China	113	Kaijin Xu et al.[2]
antivirals, corticosteroids, RRT	retrospective, single- center case series	China	138	Dawei Wang et al. [3]
RRT	case series	USA	5,700	Safiya Richardson et al. [4]
antivirals, corticosteroids, traditional Chinese medicine	retrospective	China	11	Yabo Ouyang et al. [5]
corticosteroids	letter	USA	377	Laura C. Myers et al. [6]
antivirals, corticosteroids, hydroxychloroquine, interferon, immune enhancer, thymalfasin, immunoglobulin	retrospective	China	155	Pingzheng Mo et al.[7]
antivirals, corticosteroids, immunoglobulin	prospective	China	12	Yingxia Liu et al. [8]
antivirals, interferon, corticosteroids, immunoglobulin	retrospective	China	788	Jiangshan Lian et al. [9]
anticoagulation, anti-IL6 monoclonal antibodies, antivirals, hydroxychloroquine, corticosteroids	cohort	USA	32	Jeffery P. Jacobs et al. [10]
remdesivir	cohort	USA	61	J. Grein et al. [11]
vasopressors, antivirals, hydroxychloroquine	case series	USA	24	Pavan K. Bhatraju et al. [12]
lopinavir/ritonavir, chloroquine	application note	China	22	Mingxing Huang et al. [13]
remdesivir, interferon, lopinavir/ritonavir, antibiotics, corticosteroids	randomized trials	China	237	Yeming Wang et al. [14]
antivirals, hydroxychloroquine	randomized trials	China	150	Wei Tang et al. [15]
tocilizumab, antivirals, hydroxychloroquine, cap-1002	case series	USA	34	Siddharth Singh et al. [16]
umifenovir, corticosteroids, antibiotics	retrospective	China	81	Ningfang Lian et al. [17]
antibiotics, corticosteroids, antivirals, interferon	randomized trials	China	127	Ivan Fan-Ngai Hung et al. [18]
lianhuaqingwen, antivirals, antibiotics, immunomodulator, corticosteroids	randomized trial	China	284	Ke Hu et al. [19]
hydroxychloroquine, tocilizumab, remdesivir	observational	USA	1,376	Joshua Geleris et al. [20]

hydroxychloroquine, azithromycin	non-randomized trial	France	36	Philippe Gautret et al.[21]
arbidol, lopinavir/ritonavir	cohort	China	33	Lisi Deng et al. [22]
tocilizumab, hydroxychloroquine, azithromycin, heparin, corticosteroids	retrospective	Italy	112	Marta Colaneri et al. [23]
hydroxychloroquine	pilot	China	30	CHEN Jun et al. [24]
immunoglobulin, corticosteroids, antibiotics, antivirals	cohort	China	96	Shufa Zheng et al. [25]
antivirals, antibiotics, immunoglobulin, RRT	retrospective	China	91	Xin-Ying Zhao et al. [26]
antivirals, antibiotics, corticosteroids, immunoglobulin	retrospective	China	111	Jun Zhang et al. [27]
antivirals, antibiotics, immunoglobulin, corticosteroids	observational	China	226	Yuan Yu et al. [28]
vasoconstrictive, antivirals, RRT, antibiotics, corticosteroids, immunoglobulin	observational	China	52	Xiaobo Yang et al. [29]
antivirals (lopinavir/ritonavir), antibiotics, corticosteroids vasopressor	randomized trial	China	199	B. Cao et al. [30]
antivirals, corticosteroids, immunoglobulin, RRT	cohort	China	191	Fei Zhou et al. [31]
antivirals, interferon, arbidol, antibiotics, corticosteroids, immunoglobulin	retrospective case series	China	62	Xiao-Wei Xu et al. [32]
antibiotics, antivirals, immunoglobulin, traditional Chinese medicine	descriptive	China	80	Jian Wu et al. [33]
antibiotics, antivirals, immunomodulators, antioxidant	cohort	China	201	ChaominWu et al. [34]
antivirals, antibiotics, antifungal, corticosteroids, arbidol	retrospective	China	69	Zhongliang Wang et al.[35]
antivirals, antibiotics, corticosteroids	retrospective case series	China	107	Dawei Wang et al. [36]
hydroxychloroquine, azithromycin, antivirals, tocilizumab	cross-sectional	USA	48	Lara S. Shekerdemian et al. [37]
chloroquine, hydroxychloroquine	observational	USA	201	Moussa Saleh et al. [38]
antibiotics, hydroxychloroquine, corticosteroids, antivirals, tocilizumab, vasopressors,	observational cohort	Italy	33	Simone Piva et al. [39]

RRT				
antibiotics, antivirals, antifungal, corticosteroids, interferon, immunoglobulin,	cross-sectional	China	204	Lei Pan et al. [40]
antivirals, interferon, corticosteroids, antibiotics	retrospective	Macau	10	Iek Long Lo et al. [41]
antivirals, antibiotics, immunoglobulin, thymosin, corticosteroids	retrospective	China	78	Wei Liu et al. [42]
antivirals, antibiotics, corticosteroids, immunoglobulin	retrospective	China	137	Kui Liu et al. [43]
antivirals, antibiotics, corticosteroids, immunoglobulin	retrospective	China	60	Yongpo Jiang et al. [44]
antivirals, antibiotics, corticosteroids, RRT	prospective	China	41	Chaolin Huang et al. [45]
antivirals, antibiotics, corticosteroids, RRT	retrospective	China	323	Ling Hu et al. [46]
antivirals, antibiotics, corticosteroids, RRT, hydroxychloroquine	descriptive	Korea	98	Kyung Soo Hong et al. [47]
antivirals, antibiotics, corticosteroids, RRT, antifungal, immunoglobulin	descriptive	China	99	Nanshan Chen et al. [48]
antivirals, antibiotics, corticosteroids	retrospective	China	9	Huijun Chen et al. [49]
RRT	cohort	China	102	Jianlei Cao et al. [50]
antibiotics	case series	China	10	Cai Jiehao et al. [51]
remdesivir	report	Denmark, UK, Greece, Germany , Korea, Mexico, Spain, Japan, and Singapore	1,063	J.H. Beigel et al. [52]
antibiotics	retrospective cohort	USA	89	Mohammud M. Alam et al. [53]
Antibiotics, antivirals, hydroxychloroquine	retrospective cohort	Kuwait	1,096	Sulaiman Almazeedi et al.[54]
corticosteroids, antibiotics, hydroxychloroquine, immunoglobulins	short communication	Japan	92	Nobuhiro Asai et al. [55]
hydroxychloroquine, antibiotics, tocilizumab,	retrospective observational	Turkey	109	Nijad Bakhshaliyev et al. [56]
corticosteroids, antivirals	Retrospective observational	Spain	4,035	Juan Berenguer et al. [57]
hydroxychloroquine, antivirals	retrospective	India	522	Sudhir Bhandari et al. [58]
hydroxychloroquine	randomized trial	Canada	821	D.R. Boulware et al.[59]
corticosteroids, antivirals, antibiotics, hydroxychloroquine	retrospective	USA	105	Frederick S. et al.[60]

tocilizumab	retrospective	Italy	65	Corrado,Campochiaro et al.[61]
hydroxychloroquine, antibiotics	retrospective	USA	102	Stephen Capone et al.[62]
hydroxychloroquine, antivirals, antibiotics, corticosteroids, immunoglobulins	retrospective	Spain	15,111	J.M. Casas-Rojo et al.[63]
hydroxychloroquine, antivirals, tocilizumab	retrospective	Belgium	8,910	Lucy Catteau et al.[64]
interferons, antivirals, corticosteroids	cohort	China	175	Dong Chen et al.[65]
corticosteroids, antivirals, antibiotics, chloroquine	retrospective	China	267	Xudan Chen et al.[66]
antivirals, hydroxychloroquine, tocilizumab	randomized trial	France	149	Pierre-François et al.[67]
corticosteroids, antivirals, antibiotics, hydroxychloroquine	retrospective	Italy	72	Giuseppe Di Lorenzo et al.[68]
corticosteroids, antivirals, antibiotics, chloroquine	retrospective	France	1,240	Charles Fauvel et al.[69]
corticosteroids	prospective, multicenter, cohort	Spain	663	C. Ferrando et al.[70]
hydroxychloroquine, antivirals	observational cohort	USA	11,721	Michael W. Fried et al. [71]
hydroxychloroquine, antivirals, antibiotics, corticosteroids, immunoglobulins, tocilizumab	observational	Spain	7	Alberto García-Salido et al.[72]
hydroxychloroquine, antivirals, antibiotics	retrospective	France	99	Christophe Guervilly et al. [73]
corticosteroids	cohort study	USA	2,215	Shruti Gupta et al. [74]
antivirals, hydroxychloroquine	case series	Korea	91	Mi Seon Han et al. [75]
hydroxychloroquine, tocilizumab, corticosteroids, antibiotics, antivirals	letter	Italy	26	Enrico Heffler et al. [76]
tocilizumab, corticosteroids, antibiotics, antivirals, immunoglobulins, interferons	retrospective cohort study	China	60	Mao Huang et al. [77]
antibiotics, hydroxychloroquine, antivirals	retrospective observational	USA	486	Kevin Hur et al.[78]
hydrocortisone	randomized trial	Multiple countries	403	the writing Committee for the REMAP-CAP Investigators [79]
hydroxychloroquine, antivirals	cohort	Austria	156	Mario Karolyi et al.[80]
hydroxychloroquine,	case series	USA	33	Shubhi Kaushik et al.[81]

antivirals, corticosteroids, tocilizumab				
hydroxychloroquine, antivirals, corticosteroids, tocilizumab, interferons	cohort	Oman	63	Faryal Khamis et al. [82]
hydroxychloroquine, antivirals, antibiotics,	report	USA	576	Lindsay Kim et al. [83]
RRT	observational	Korea	1,082	Mi Kyung Kim et al. [84]
hydroxychloroquine, antibiotics, antivirals, corticosteroids	retrospective case-control	USA	135	Issam Koleilat et al. [85]
corticosteroids	correspondence	USA	152	Sandeep Krishna et al.[86]
hydroxychloroquine, antivirals	report	France	80	Marie Lecronier et al.[87]
Antivirals, hydroxychloroquine, corticosteroids	retrospective	Korea	98	Ji Yeon Lee et al. [88]
antivirals, interferons, hydroxychloroquine	cohort	Singapore	201	Pei Hua Lee et al.[89]
RRT	retrospective	North Zealand, Denmark	115	Maria Elisabeth Lendorf et al. [90]
antivirals, antibiotics, corticosteroids, interferons, antifungal, immunoglobulins	cohort	China	3,375	Liyang Li et al.[91]
antivirals, corticosteroids	randomized trial	China	86	Yueping Li et al.[92]
corticosteroids	retrospective observational	France	268	Sophie Liabeuf et al. [93]
corticosteroids, RRT	report	China	65	fangfang Liu et al. [94]
antivirals, antibiotics hydroxychloroquine,	retrospective	USA	46	Erica M. Lokken et al. [95]
hydroxychloroquine, corticosteroids	letter	Italy	121	Andrea Lovato et al. [96]
hydroxychloroquine, antibiotics, corticosteroids, tocilizumab	retrospective	USA	224	Tetsuro Maeda et al.[97]
hydroxychloroquine, azithromycin	cohort	Iran	35	Shima Mahmoudi et al.[98]
dexamethasone	randomized trial	UK	11,303	The RECOVERY Collaborative Group [99]
hydroxychloroquine, antibiotics	retrospective cohort	USA	6,493	Takahisa Mikami et al.[100]
hydroxychloroquine, antivirals	observational	Iran	22	Ali Monfared et al. [101]
corticosteroids	letter	France	96	Francois Montastruc et al. [102]
hydroxychloroquine, antivirals, corticosteroids, antibiotics, tocilizumab	retrospective observational	Spain	62	Fernando Montero et al. [103]
tocilizumab, hydroxychloroquine, antivirals, antibiotics	prospective	Italy	51	Valentina Morena et al. [104]
tocilizumab	retrospective cohort	Spain	236	Oscar Moreno-Pérez et al.[105]

Tocilizumab, hydroxychloroquine, antivirals, corticosteroids, RRT	retrospective cohort	USA	81	Austin R. Morrison et al.[106]
corticosteroids	retrospective cohort	Spain	404	Jose M. Mostaza et al. [107]
tocilizumab, hydroxychloroquine, antivirals, antibiotics, corticosteroids	retrospective cohort	Paris	279	Yann Nguyen et al.[108]
RRT	retrospective	Poland	169	Błażej Nowak et al.[109]
antivirals, chloroquine, antibiotics, corticosteroids	retrospective cohort	USA	251	Alexis K. Okoh et al.[110]
antivirals, antibiotics, corticosteroids	report	Italy	3,032	Luigi Palmieri et al.[111]
antivirals, hydroxychloroquine, tocilizumab	cohort	Italy	51	Zeno Pasquini et al.[112]
corticosteroids, hydroxychloroquine, tocilizumab	retrospective cohort	USA	223	Jerald Pelayo et al.[113]
antivirals	retrospective	Switzerland	196	Pellaud Charlotte et al. [114]
corticosteroids, hydroxychloroquine, tocilizumab, antivirals	retrospective	Italy	10	Nicola Potere et al.[115]
hydroxychloroquine, corticosteroids, antivirals	observational	USA	239	Christina C. Price et al.[60]
hydroxychloroquine, antivirals antibiotics,	Prospective observational	Switzerland	129	Steve Primmaz et al. [116]
corticosteroids, hydroxychloroquine, tocilizumab, antivirals	retrospective	Italy	111	Luca Quartuccio et al.[117]
hydroxychloroquine, antibiotics	retrospective	USA	1,438	Eli S. Rosenberg et al. [118]
corticosteroids, hydroxychloroquine, tocilizumab	cohort	USA	242	Grace Salacup et al. [119]
hydroxychloroquine, antibiotics, antivirals, corticosteroids	retrospective	France	54	Loic Sentilhes et al. [120]
hydroxychloroquine, antibiotics, antivirals, corticosteroids	cohort	USA	186	Sachin J. Shah et al. [121]
antibiotics, antivirals, corticosteroids, immunoglobulin	retrospective	China	99	Ding Shi et al. et al.[122]
hydroxychloroquine	randomized trial	USA and Canada	491	Caleb P. Skipper et al.[123]
hydroxychloroquine, antibiotics, antivirals, corticosteroids, tocilizumab	randomized trial	US, Europe, and Asia	596	Christoph D. Spinner et al.[124]
antibiotics, RRT	Case series	USA	463	Geehan Suleyman et al. [125]
hydroxychloroquine, antibiotics, antivirals, corticosteroids	randomized trial	Brazil	299	Bruno M. Tomazini et al.[126]

tocilizumab	prospective series	Italy	100	Paola Toniati et al.[127]
hydroxychloroquine, antivirals	letter	USA	2,130	Farhaan S. Vahidy et al. [128]
corticosteroids	retrospective	China	1,514	Jianfeng Wu et al. [129]
antivirals	cohort	China	146	Ping Xu et al. [130]
antivirals, antibiotics, corticosteroids	retrospective	China	120	Dan Yan et al. [131]
antivirals, antibiotics, RRT, interferons, corticosteroids	retrospective	China	218	Xiquan Yan et al. [132]
hydroxychloroquine, antivirals, corticosteroids	retrospective	France	821	Lorène Zerah et al.[133]
RRT, corticosteroids, antibiotics	retrospective observational	China	74	Qianhui Zhang et al. [134]
corticosteroids, antibiotics, antivirals	retrospective	China	258	Yan Zhang et al.[135]
antivirals	retrospective	China	50	Zhen Zhu et al. [136]

Note: RRT, renal replacement therapy.

Supplementary Table 3. Heterogeneity results from the proportional meta-analysis

**Treatment**	**τ^2 [95% CI]**	**I^2 [95% CI]**	**H^2 [95% CI]**	**Q test (*p*-value)**
antibiotics	0.15 [0.11, 0.22]	99.56 [99.39, 99.69]	225.5 [164.2, 328.9]	15812.98 (< 0.0001)
Corticosteroids	0.065 [0.047, 0.094]	99.26 [98.98, 99.49]	136.9 [98.43 197.6]	6277.3 (< 0.0001)
antivirals	0.17 [0.13, 0.23]	99.69 [99.6, 99.77]	328.34 [250.86, 442.91]	30492.92 (< 0.0001)
chloroquine or hydroxychloroquine	0.15 [0.12, 0.22]	99.69 [99.56, 99.79]	323.80 [231.51, 478.91]	10863.67 (< 0.0001)
immunoglobulin	0.035 [0.019, 0.073]	96.61 [94.12, 98.36]	29.53 [17.00, 60.98]	604.05 (< 0.0001)
interferon	0.16 [0.087, 0.39]	99.53 [99.14, 99.8]	216.6 [116.59, 523.38]	2115.89 (< 0.0001)
RRT	0.021 [0.01, 0.05]	95.91 [92.99, 98.12]	24.5 [14.27, 53.36]	305.68 (< 0.0001)
tocilizumab	0.10 [0.06, 0.19]	99.72 [99.54, 99.84]	358.49 [220.29, 663.69]	3910.91 (< 0.0001)

Note: τ^2, estimated amount of total heterogeneity, I^2, total heterogeneity / total variability, H^2, total variability / sampling variability, Q test, overall test of heterogeneity.

Supplementary Table 4. A summary of the 20 studies included in the network meta-analysis

Experimental group	Control group	Study
lopinavir/ritonavir	standard care	B. Cao et al. [30]
lopinavir/ritonavir + interferon	lopinavir/ritonavir	Ivan Fan-Ngai Hung et al.[18]
remdesivir	standard care	Yeming Wang et al. [14]
hydroxychloroquine	standard care	Wei Tang et al.[15]
hydroxychloroquine	standard care	Joshua Geleris et al. [20]
remdesivir	standard care	J.H. Beigel et al. [52]
arbidol+lopinavir/ ritonavir	lopinavir/ritonavir	Lisi Deng et al. [22]
chloroquine	lopinavir/ritonavir	Mingxing Huang et al. [13]
hydroxychloroquine	standard care	Philippe Gautret et al. [21]
umifenovir	standard care	Ningfang Lian et al. [17]
lopinavir/ritonavir	arbidol	Zhen Zhu et al. [136]
hydroxychloroquine/ chloroquine	lopinavir/ritonavir	Marie Lecronier et al. [87]
dexamethasone	standard care	The RECOVERY Collaborative Group [99]
dexamethasone	standard care	Bruno M. Tomazini et al.[126]
hydrocortisone	standard care	Pierre-Francois Dequin et al [67]
hydrocortisone	standard care	the writing Committee for the R EMAP-CAP Investigators [79]
remedesivir	standard care	Christoph D. Spinner et al [124]
hydroxychloroquine/ chloroquine	standard care	Caleb P. Skipper et al.[123]

Hydroxychloroquine/ Chloroquine	Lopinavir/ritonavir	Mario Karolyi et al.[80]
Corticosteroids	standard care	Jianfeng Wu et al [129].

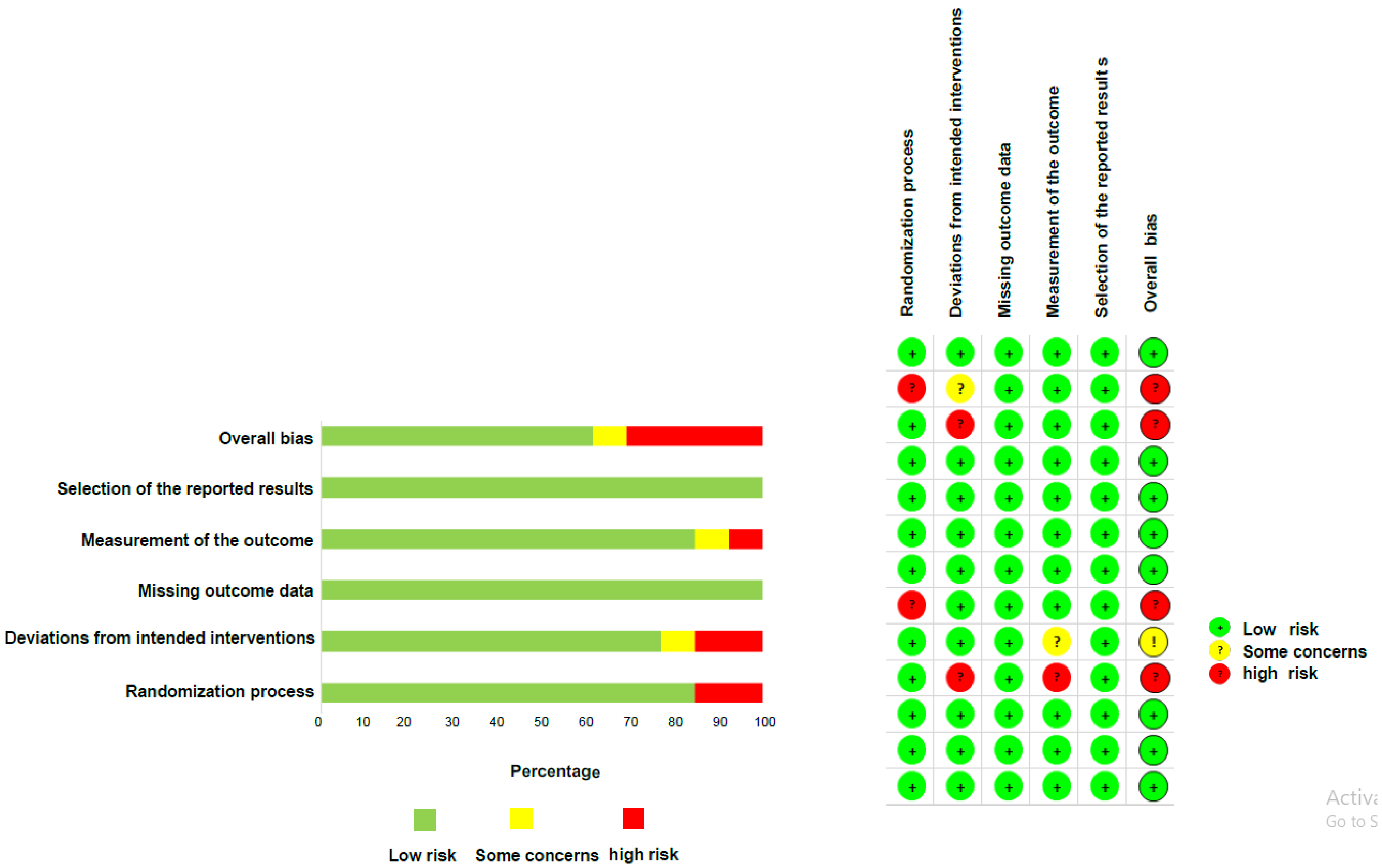

Supplementary Figure 1. Cochrane risk of bias analysis (ROB-2) of 13 randomized trial studies of COVID-19 treatment

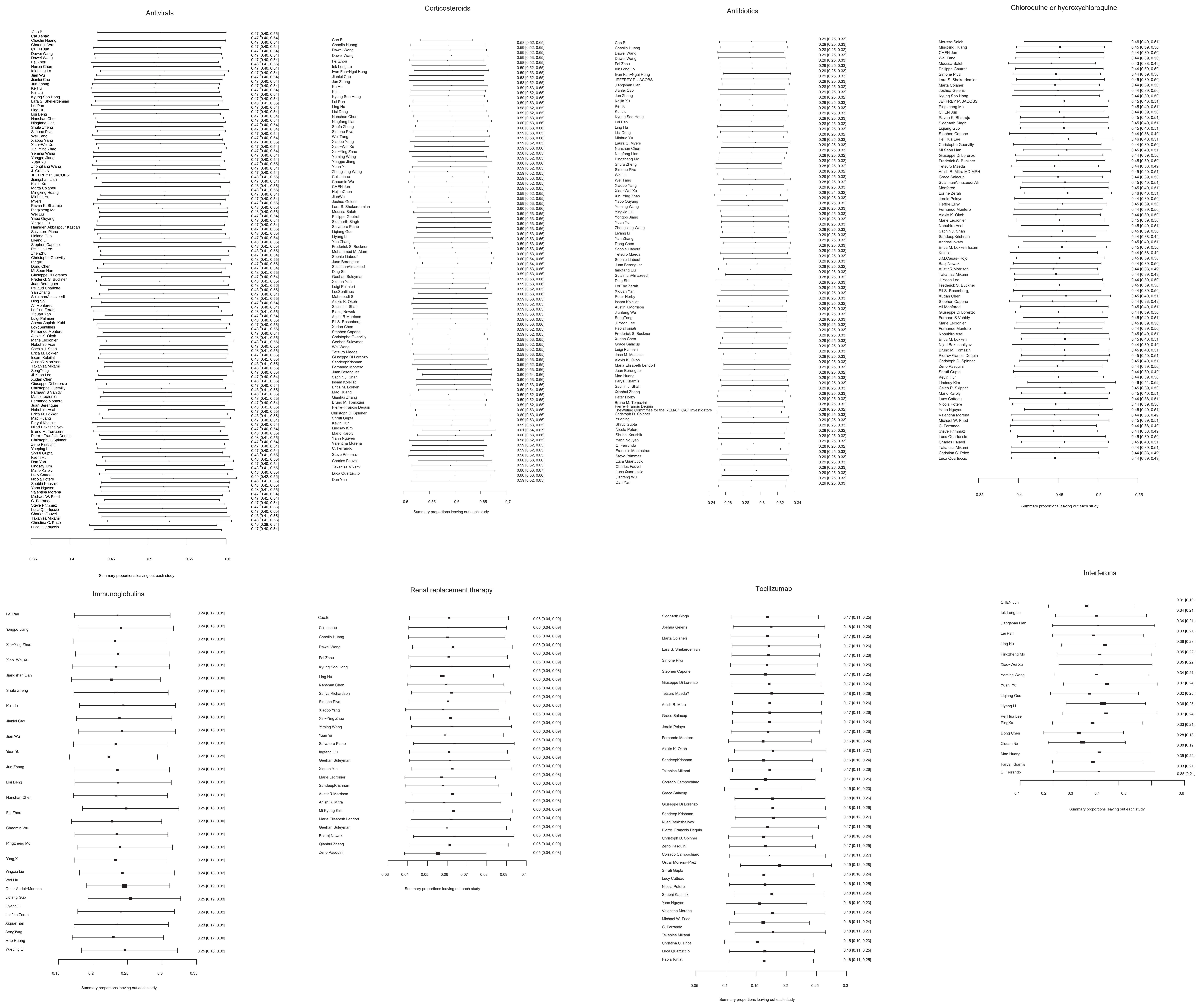

Supplementary Figure 2. Forest plots showing outlying and influential studies in eight treatments by externally studentized residuals analysis. Each box represents a summary proportion estimated by the leave-one-out study.

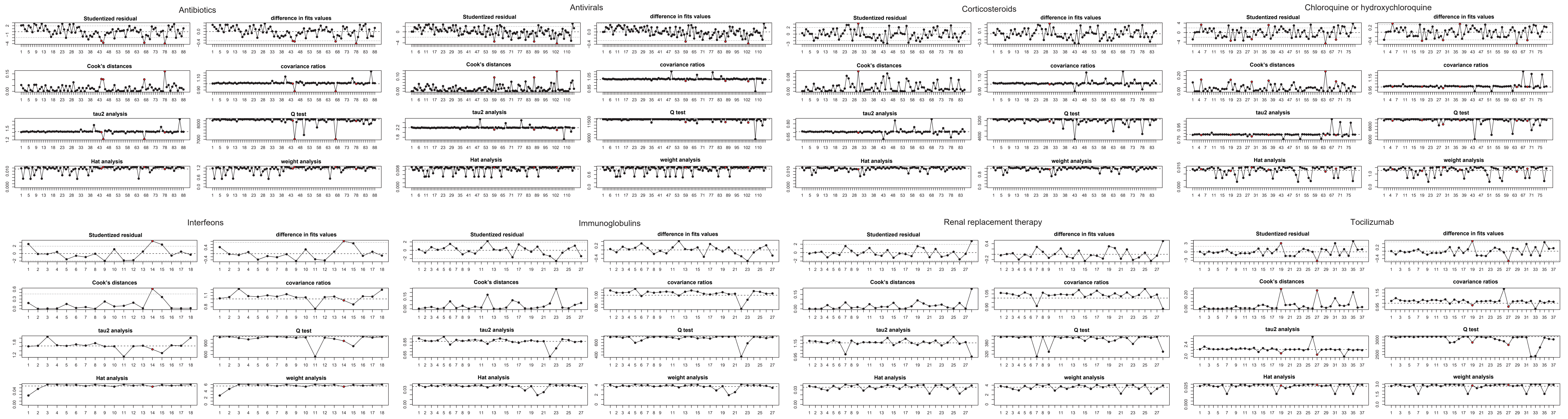

Supplementary Figure 3. Diagnostic plots showing significant and influential studies by analyses of externally studentized residuals, difference in fits values, Cook's distances, covariance ratios, tau2 ,and Q-test of leave-one-out heterogeneity, hat values, and weights.

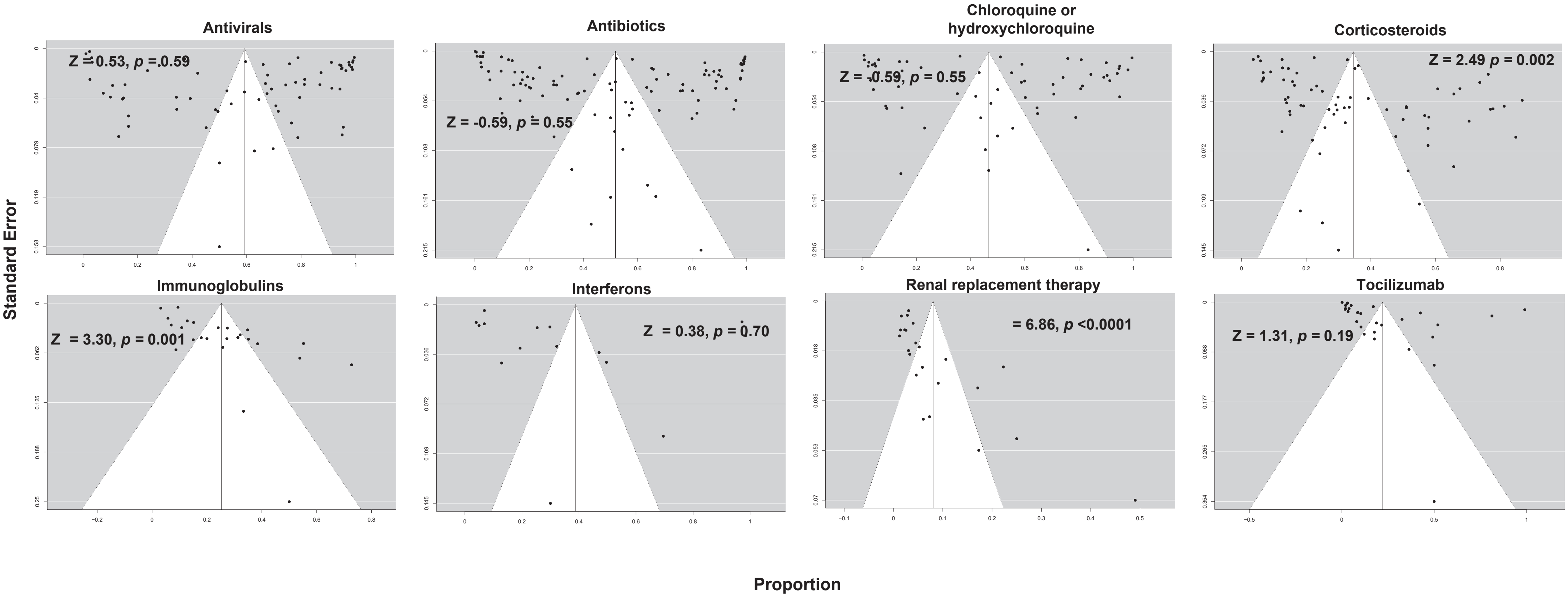

Supplementary Figure 4. Funnel plots showing proportional meta-analysis publication bias by testing for the funnel plot asymmetry using the Egger's regression test. The Z and P values are shown.

References

1. Yu M, Liu Y, Xu D, Zhang R, Lan L, Xu H. Prediction of the Development of Pulmonary Fibrosis Using Serial Thin-Section CT and Clinical Features in Patients Discharged after Treatment for COVID-19 Pneumonia. *Korean journal of radiology.* 2020;21(6):746-755.
2. Xu K, Chen Y, Yuan J, et al. Factors associated with prolonged viral RNA shedding in patients with COVID-19. *Clinical infectious diseases : an official publication of the Infectious Diseases Society of America.* 2020.
3. Wang D, Hu B, Hu C, et al. Clinical Characteristics of 138 Hospitalized Patients With 2019 Novel Coronavirus-Infected Pneumonia in Wuhan, China. *Jama.* 2020.
4. Richardson S, Hirsch JS, Narasimhan M, et al. Presenting Characteristics, Comorbidities, and Outcomes Among 5700 Patients Hospitalized With COVID-19 in the New York City Area. *Jama.* 2020.
5. Ouyang Y, Yin J, Wang W, et al. Down-regulated gene expression spectrum and immune responses changed during the disease progression in COVID-19 patients. *Clinical infectious diseases : an official publication of the Infectious Diseases Society of America.* 2020.
6. Myers LC, Parodi SM, Escobar GJ, Liu VX. Characteristics of Hospitalized Adults With COVID-19 in an Integrated Health Care System in California. *Jama.* 2020.
7. Mo P, Xing Y, Xiao Y, et al. Clinical characteristics of refractory COVID-19 pneumonia in Wuhan, China. *Clinical infectious diseases : an official publication of the Infectious Diseases Society of America.* 2020.
8. Liu Y, Yang Y, Zhang C, et al. Clinical and biochemical indexes from 2019-nCoV infected patients linked to viral loads and lung injury. *Science China Life sciences.* 2020;63(3):364-374.
9. Lian J, Jin X, Hao S, et al. Analysis of Epidemiological and Clinical features in older patients with Corona Virus Disease 2019 (COVID-19) out of Wuhan. *Clinical infectious diseases : an official publication of the Infectious Diseases Society of America.* 2020.
10. Jacobs JP, Stammers AH, St Louis J, et al. Extracorporeal Membrane Oxygenation in the Treatment of Severe Pulmonary and Cardiac Compromise in COVID-19: Experience with 32 patients. *ASAIO journal (American Society for Artificial Internal Organs : 1992).* 2020.
11. Grein J, Ohmagari N, Shin D, et al. Compassionate Use of Remdesivir for Patients with Severe Covid-19. *New England Journal of Medicine.* 2020.
12. Bhatraju PK, Ghassemieh BJ, Nichols M, et al. Covid-19 in Critically Ill Patients in the Seattle Region — Case Series. *New England Journal of Medicine.* 2020.
13. Huang M, Tang T, Pang P, et al. Treating COVID-19 with Chloroquine. *Journal of molecular cell biology.* 2020;12(4):322-325.
14. Wang Y, Zhang D, Du G, et al. Remdesivir in adults with severe COVID-19: a randomised, double-blind, placebo-controlled, multicentre trial. *The Lancet.* 2020.
15. Tang W, Cao Z, Han M, et al. Hydroxychloroquine in patients with mainly mild to moderate coronavirus disease 2019: open label, randomised controlled trial. *BMJ (Clinical research ed).* 2020;369:m1849.
16. Singh S, Chakravarty T, Chen P, et al. Allogeneic cardiosphere-derived cells (CAP-1002) in critically ill COVID-19 patients: compassionate-use case series. *Basic research in cardiology.* 2020;115(4):36.
17. Lian N, Xie H, Lin S, Huang J, Zhao J, Lin Q. Umifenovir treatment is not associated with improved outcomes in patients with coronavirus disease 2019: a retrospective study. *Clinical microbiology and infection : the official publication of the European Society of Clinical Microbiology and Infectious Diseases.* 2020.
18. Hung IF, Lung KC, Tso EY, et al. Triple combination of interferon beta-1b, lopinavir-ritonavir, and ribavirin in the treatment of patients admitted to hospital with COVID-19: an open-label, randomised, phase 2 trial. *Lancet (London, England).* 2020.

19. Hu K, Guan WJ, Bi Y, et al. Efficacy and Safety of Lianhuaqingwen Capsules, a repurposed Chinese Herb, in Patients with Coronavirus disease 2019: A multicenter, prospective, randomized controlled trial. *Phytomedicine.* 2020:153242.
20. Geleris J, Sun Y, Platt J, et al. Observational Study of Hydroxychloroquine in Hospitalized Patients with Covid-19. *New England Journal of Medicine.* 2020.
21. Gautret P, Lagier JC, Parola P, et al. Hydroxychloroquine and azithromycin as a treatment of COVID-19: results of an open-label non-randomized clinical trial. *International journal of antimicrobial agents.* 2020:105949.
22. Deng L, Li C, Zeng Q, et al. Arbidol combined with LPV/r versus LPV/r alone against Corona Virus Disease 2019: A retrospective cohort study. *The Journal of infection.* 2020.
23. Colaneri M, Bogliolo L, Valsecchi P, et al. Tocilizumab for Treatment of Severe COVID-19 Patients: Preliminary Results from SMAtteo COvid19 REgistry (SMACORE). *Microorganisms.* 2020;8(5).
24. CHEN Jun LD, LIU Li,LIU Ping,XU Qingnian,XIA Lu,LING Yun,HUANG Dan,SONG Shuli,ZHANG Dandan,QIAN Zhiping,LI Tao,SHEN Yinzhong,LU Hongzhou. A pilot study of hydroxychloroquine in treatment of patients with common coronavirus disease-19 (COVID-19). *J Zhejiang Univ (Med Sci).* 2020;49(1):0-0.
25. Zheng S, Fan J, Yu F, et al. Viral load dynamics and disease severity in patients infected with SARS-CoV-2 in Zhejiang province, China, January-March 2020: retrospective cohort study. *BMJ (Clinical research ed).* 2020;369:m1443.
26. Zhao XY, Xu XX, Yin HS, et al. Clinical characteristics of patients with 2019 coronavirus disease in a non-Wuhan area of Hubei Province, China: a retrospective study. *BMC infectious diseases.* 2020;20(1):311.
27. Zhang J, Yu M, Tong S, Liu LY, Tang LV. Predictive factors for disease progression in hospitalized patients with coronavirus disease 2019 in Wuhan, China. *Journal of clinical virology : the official publication of the Pan American Society for Clinical Virology.* 2020;127:104392.
28. Yu Y, Xu D, Fu S, et al. Patients with COVID-19 in 19 ICUs in Wuhan, China: a cross-sectional study. *Critical care (London, England).* 2020;24(1):219.
29. Yang X, Yu Y, Xu J, et al. Clinical course and outcomes of critically ill patients with SARS-CoV-2 pneumonia in Wuhan, China: a single-centered, retrospective, observational study. *The Lancet Respiratory medicine.* 2020.
30. Cao B, Wang Y, Wen D, et al. A Trial of Lopinavir-Ritonavir in Adults Hospitalized with Severe Covid-19. *The New England journal of medicine.* 2020.
31. Zhou F, Yu T, Du R, et al. Clinical course and risk factors for mortality of adult inpatients with COVID-19 in Wuhan, China: a retrospective cohort study. *Lancet (London, England).* 2020;395(10229):1054-1062.
32. Xu XW, Wu XX, Jiang XG, et al. Clinical findings in a group of patients infected with the 2019 novel coronavirus (SARS-Cov-2) outside of Wuhan, China: retrospective case series. *BMJ (Clinical research ed).* 2020;368:m606.
33. Wu J, Liu J, Zhao X, et al. Clinical Characteristics of Imported Cases of COVID-19 in Jiangsu Province: A Multicenter Descriptive Study. *Clinical infectious diseases : an official publication of the Infectious Diseases Society of America.* 2020.
34. Wu C, Chen X, Cai Y, et al. Risk Factors Associated With Acute Respiratory Distress Syndrome and Death in Patients With Coronavirus Disease 2019 Pneumonia in Wuhan, China. *JAMA internal medicine.* 2020.
35. Wang Z, Yang B, Li Q, Wen L, Zhang R. Clinical Features of 69 Cases with Coronavirus Disease 2019 in Wuhan, China. *Clinical infectious diseases : an official publication of the Infectious Diseases Society of America.* 2020.
36. Wang D, Yin Y, Hu C, et al. Clinical course and outcome of 107 patients infected with the novel coronavirus, SARS-CoV-2, discharged from two hospitals in Wuhan, China. *Critical care (London, England).* 2020;24(1):188.
37. Shekerdemian LS, Mahmood NR, Wolfe KK, et al. Characteristics and Outcomes of Children With Coronavirus Disease 2019 (COVID-19) Infection Admitted to US and

Canadian Pediatric Intensive Care Units. *JAMA Pediatrics.* 2020.
38. Saleh M, Gabriels J, Chang D, et al. The Effect of Chloroquine, Hydroxychloroquine and Azithromycin on the Corrected QT Interval in Patients with SARS-CoV-2 Infection. *Circulation Arrhythmia and electrophysiology.* 2020.
39. Piva S, Filippini M, Turla F, et al. Clinical presentation and initial management critically ill patients with severe acute respiratory syndrome coronavirus 2 (SARS-CoV-2) infection in Brescia, Italy. *Journal of critical care.* 2020;58:29-33.
40. Pan L, Mu M, Yang P, et al. Clinical Characteristics of COVID-19 Patients With Digestive Symptoms in Hubei, China: A Descriptive, Cross-Sectional, Multicenter Study. *The American journal of gastroenterology.* 2020;115(5):766-773.
41. Lo IL, Lio CF, Cheong HH, et al. Evaluation of SARS-CoV-2 RNA shedding in clinical specimens and clinical characteristics of 10 patients with COVID-19 in Macau. *International journal of biological sciences.* 2020;16(10):1698-1707.
42. Liu W, Tao Z-W, Wang L, et al. Analysis of factors associated with disease outcomes in hospitalized patients with 2019 novel coronavirus disease. *Chinese Medical Journal.* 2020;133(9):1032-1038.
43. Liu K, Fang Y-Y, Deng Y, et al. Clinical characteristics of novel coronavirus cases in tertiary hospitals in Hubei Province. *Chinese medical journal.* 2020:10.1097/CM1099.0000000000000744.
44. Jiang Y, He S, Zhang C, et al. Clinical characteristics of 60 discharged cases of 2019 novel coronavirus-infected pneumonia in Taizhou, China. *Annals of translational medicine.* 2020;8(8):547.
45. Huang C, Wang Y, Li X, et al. Clinical features of patients infected with 2019 novel coronavirus in Wuhan, China. *Lancet (London, England).* 2020;395(10223):497-506.
46. Hu L, Chen S, Fu Y, et al. Risk Factors Associated with Clinical Outcomes in 323 COVID-19 Hospitalized Patients in Wuhan, China. *Clinical infectious diseases : an official publication of the Infectious Diseases Society of America.* 2020.
47. Hong KS, Lee KH, Chung JH, et al. Clinical Features and Outcomes of 98 Patients Hospitalized with SARS-CoV-2 Infection in Daegu, South Korea: A Brief Descriptive Study. *Yonsei medical journal.* 2020;61(5):431-437.
48. Chen N, Zhou M, Dong X, et al. Epidemiological and clinical characteristics of 99 cases of 2019 novel coronavirus pneumonia in Wuhan, China: a descriptive study. *Lancet (London, England).* 2020;395(10223):507-513.
49. Chen H, Guo J, Wang C, et al. Clinical characteristics and intrauterine vertical transmission potential of COVID-19 infection in nine pregnant women: a retrospective review of medical records. *The Lancet.* 2020;395(10226):809-815.
50. Cao J, Tu WJ, Cheng W, et al. Clinical Features and Short-term Outcomes of 102 Patients with Corona Virus Disease 2019 in Wuhan, China. *Clinical infectious diseases : an official publication of the Infectious Diseases Society of America.* 2020.
51. Cai J, Xu J, Lin D, et al. A Case Series of children with 2019 novel coronavirus infection: clinical and epidemiological features. *Clinical infectious diseases : an official publication of the Infectious Diseases Society of America.* 2020:ciaa198.
52. Beigel JH, Tomashek KM, Dodd LE, et al. Remdesivir for the Treatment of Covid-19 - Preliminary Report. *The New England journal of medicine.* 2020.
53. Alam MM, Mahmud S, Rahman MM, Simpson J, Aggarwal S, Ahmed Z. Clinical Outcomes of Early Treatment With Doxycycline for 89 High-Risk COVID-19 Patients in Long-Term Care Facilities in New York. *Cureus.* 2020;12(8):e9658.
54. Almazeedi S, Al-Youha S, Jamal MH, et al. Characteristics, risk factors and outcomes among the first consecutive 1096 patients diagnosed with COVID-19 in Kuwait. *EClinicalMedicine.* 2020;24:100448.
55. Asai N, Sakanashi D, Nakamura A, et al. Clinical manifestations and radiological features by chest computed tomographic findings of a novel coronavirus disease-19 pneumonia among 92 patients in Japan. *Journal of microbiology, immunology, and infection = Wei mian yu gan ran za zhi.* 2020.
56. Bakhshaliyev N, Uluganyan M, Enhos A, Karacop E, Ozdemir R. The effect of 5-day

course of hydroxychloroquine and azithromycin combination on QT interval in non-ICU COVID19(+) patients. *Journal of electrocardiology.* 2020;62:59-64.
57. Berenguer J, Ryan P, Rodríguez-Baño J, et al. Characteristics and predictors of death among 4,035 consecutively hospitalized patients with COVID-19 in Spain. *Clinical microbiology and infection : the official publication of the European Society of Clinical Microbiology and Infectious Diseases.* 2020.
58. Bhandari S, Singh A, Sharma R, et al. Characteristics, Treatment Outcomes and Role of Hydroxychloroquine among 522 COVID-19 hospitalized patients in Jaipur City: An Epidemio-Clinical Study. *The Journal of the Association of Physicians of India.* 2020;68(6):13-19.
59. Boulware DR, Pullen MF, Bangdiwala AS, et al. A Randomized Trial of Hydroxychloroquine as Postexposure Prophylaxis for Covid-19. 2020.
60. Price CC, Altice FL, Shyr Y, et al. Tocilizumab treatment for cytokine release syndrome in hospitalized COVID-19 patients: survival and clinical outcomes. 2020.
61. Campochiaro C, Della-Torre E, Cavalli G, et al. Efficacy and safety of tocilizumab in severe COVID-19 patients: a single-centre retrospective cohort study. *European journal of internal medicine.* 2020;76:43-49.
62. Capone S, Abramyan S, Ross B, et al. Characterization of Critically Ill COVID-19 Patients at a Brooklyn Safety-Net Hospital. *Cureus.* 2020;12(8):e9809.
63. Casas-Rojo JM, Antón-Santos JM, Millán-Núñez-Cortés J, et al. Clinical characteristics of patients hospitalized with COVID-19 in Spain: Results from the SEMI-COVID-19 Registry. *Revista clinica espanola.* 2020.
64. Catteau L, Dauby N, Montourcy M, et al. Low-dose Hydroxychloroquine Therapy and Mortality in Hospitalized Patients with COVID-19: A Nationwide Observational Study of 8075 Participants. *International journal of antimicrobial agents.* 2020:106144.
65. Chen D, Li X, Song Q, et al. Assessment of Hypokalemia and Clinical Characteristics in Patients With Coronavirus Disease 2019 in Wenzhou, China. *JAMA Network Open.* 2020;3(6):e2011122-e2011122.
66. Chen X, Zhu B, Hong W, et al. Associations of clinical characteristics and treatment regimens with the duration of viral RNA shedding in patients with COVID-19. *International journal of infectious diseases : IJID : official publication of the International Society for Infectious Diseases.* 2020;98:252-260.
67. Dequin P-F, Heming N, Meziani F, et al. Effect of Hydrocortisone on 21-Day Mortality or Respiratory Support Among Critically Ill Patients With COVID-19: A Randomized Clinical Trial. *Jama.* 2020.
68. Di Lorenzo G, Buonerba L, Ingenito C, et al. Clinical Characteristics of Metastatic Prostate Cancer Patients Infected with COVID-19 in South Italy. *Oncology.* 2020:1-5.
69. Fauvel C, Weizman O, Trimaille A, et al. Pulmonary embolism in COVID-19 patients: a French multicentre cohort study. *European heart journal.* 2020;41(32):3058-3068.
70. Ferrando C, Mellado-Artigas R, Gea A, et al. Patient characteristics, clinical course and factors associated to ICU mortality in critically ill patients infected with SARS-CoV-2 in Spain: A prospective, cohort, multicentre study. *Revista espanola de anestesiologia y reanimacion.* 2020.
71. Fried MW, Crawford JM, Mospan AR, et al. Patient Characteristics and Outcomes of 11,721 Patients with COVID19 Hospitalized Across the United States. *Clinical infectious diseases : an official publication of the Infectious Diseases Society of America.* 2020.
72. García-Salido A, Leoz-Gordillo I, Martínez de Azagra-Garde A, et al. Children in Critical Care Due to Severe Acute Respiratory Syndrome Coronavirus 2 Infection: Experience in a Spanish Hospital. *Pediatric critical care medicine : a journal of the Society of Critical Care Medicine and the World Federation of Pediatric Intensive and Critical Care Societies.* 2020;21(8):e576-e580.
73. Guervilly C, Burtey S, Sabatier F, et al. Circulating Endothelial Cells as a Marker of Endothelial Injury in Severe COVID -19. *The Journal of infectious diseases.* 2020.
74. Gupta S, Hayek SS, Wang W, et al. Factors Associated With Death in Critically Ill

Patients With Coronavirus Disease 2019 in the US. *JAMA internal medicine.* 2020.
75. Han MS, Choi EH, Chang SH, et al. Clinical Characteristics and Viral RNA Detection in Children With Coronavirus Disease 2019 in the Republic of Korea. *JAMA Pediatr.* 2020.
76. Heffler E, Detoraki A, Contoli M, et al. COVID-19 in Severe Asthma Network in Italy (SANI) patients: Clinical features, impact of comorbidities and treatments. *Allergy.* 2020.
77. Huang M, Yang Y, Shang F, et al. Clinical Characteristics and Predictors of Disease Progression in Severe Patients with COVID-19 Infection in Jiangsu Province, China: A Descriptive Study. *The American journal of the medical sciences.* 2020;360(2):120-128.
78. Hur K, Price CPE, Gray EL, et al. Factors Associated With Intubation and Prolonged Intubation in Hospitalized Patients With COVID-19. *Otolaryngology--head and neck surgery : official journal of American Academy of Otolaryngology-Head and Neck Surgery.* 2020;163(1):170-178.
79. Investigators TWCftR-C. Effect of Hydrocortisone on Mortality and Organ Support in Patients With Severe COVID-19: The REMAP-CAP COVID-19 Corticosteroid Domain Randomized Clinical Trial. *Jama.* 2020.
80. Karolyi M, Pawelka E, Mader T, et al. Hydroxychloroquine versus lopinavir/ritonavir in severe COVID-19 patients : Results from a real-life patient cohort. *Wiener klinische Wochenschrift.* 2020:1-8.
81. Kaushik S, Aydin SI, Derespina KR, et al. Multisystem Inflammatory Syndrome in Children Associated with Severe Acute Respiratory Syndrome Coronavirus 2 Infection (MIS-C): A Multi-institutional Study from New York City. *The Journal of pediatrics.* 2020;224:24-29.
82. Khamis F, Al-Zakwani I, Al Naamani H, et al. Clinical characteristics and outcomes of the first 63 adult patients hospitalized with COVID-19: An experience from Oman. *Journal of infection and public health.* 2020;13(7):906-913.
83. Kim L, Whitaker M, O'Halloran A, et al. Hospitalization Rates and Characteristics of Children Aged< 18 Years Hospitalized with Laboratory-Confirmed COVID-19—COVID-NET, 14 States, March 1–July 25, 2020. 2020;69(32):1081.
84. Kim MK, Jeon JH, Kim SW, et al. The Clinical Characteristics and Outcomes of Patients with Moderate-to-Severe Coronavirus Disease 2019 Infection and Diabetes in Daegu, South Korea. *Diabetes & metabolism journal.* 2020;44(4):602-613.
85. Koleilat I, Galen B, Choinski K, et al. Clinical characteristics of acute lower extremity deep venous thrombosis diagnosed by duplex in patients hospitalized for coronavirus disease 2019. *Journal of vascular surgery Venous and lymphatic disorders.* 2020.
86. Krishnan S, Patel K, Desai R, et al. Clinical comorbidities, characteristics, and outcomes of mechanically ventilated patients in the State of Michigan with SARS-CoV-2 pneumonia. *Journal of clinical anesthesia.* 2020;67:110005.
87. Lecronier M, Beurton A, Burrel S, et al. Comparison of hydroxychloroquine, lopinavir/ritonavir, and standard of care in critically ill patients with SARS-CoV-2 pneumonia: an opportunistic retrospective analysis. *Critical care (London, England).* 2020;24(1):418.
88. Lee JY, Kim HA, Huh K, et al. Risk Factors for Mortality and Respiratory Support in Elderly Patients Hospitalized with COVID-19 in Korea. *Journal of Korean medical science.* 2020;35(23):e223.
89. Lee PH, Tay WC, Sutjipto S, et al. Associations of viral ribonucleic acid (RNA) shedding patterns with clinical illness and immune responses in Severe Acute Respiratory Syndrome Coronavirus 2 (SARS-CoV-2) infection. *Clinical & translational immunology.* 2020;9(7):e1160.
90. Lendorf ME, Boisen MK, Kristensen PL, et al. Characteristics and early outcomes of patients hospitalised for COVID-19 in North Zealand, Denmark. *Danish medical journal.* 2020;67(9).
91. Li L, Huang Q, Wang DC, Ingbar DH, Wang XJC, Medicine T. Acute lung injury in

patients with COVID-19 infection. 2020;10(1):20-27.
92. Li Y, Xie Z, Lin W, et al. Efficacy and Safety of Lopinavir/Ritonavir or Arbidol in Adult Patients with Mild/Moderate COVID-19: An Exploratory Randomized Controlled Trial. *Med (New York, NY).* 2020.
93. Liabeuf S, Moragny J, Bennis Y, et al. Association between renin-angiotensin system inhibitors and COVID-19 complications. *European heart journal Cardiovascular pharmacotherapy.* 2020.
94. Liu F, Ji C, Luo J, et al. Clinical characteristics and corticosteroids application of different clinical types in patients with corona virus disease 2019. *Scientific reports.* 2020;10(1):13689.
95. Lokken EM, Walker CL, Delaney S, et al. Clinical characteristics of 46 pregnant women with a severe acute respiratory syndrome coronavirus 2 infection in Washington State. *American journal of obstetrics and gynecology.* 2020.
96. Lovato A, Galletti C, Galletti B, de Filippis C. Clinical characteristics associated with persistent olfactory and taste alterations in COVID-19: A preliminary report on 121 patients. *American journal of otolaryngology.* 2020;41(5):102548.
97. Maeda T, Obata R, Rizk DD, Kuno T. The association of interleukin-6 value, interleukin inhibitors, and outcomes of patients with COVID-19 in New York City. *Journal of medical virology.* 2020.
98. Mahmoudi S, Mehdizadeh M, Shervin Badv R, et al. The Coronavirus Disease 2019 (COVID-19) in Children: A Study in an Iranian Children's Referral Hospital. *Infection and drug resistance.* 2020;13:2649-2655.
99. Medicine RCGJNEJo. Dexamethasone in Hospitalized Patients with Covid-19—Preliminary Report. 2020.
100. Mikami T, Miyashita H, Yamada T, et al. Risk Factors for Mortality in Patients with COVID-19 in New York City. *Journal of general internal medicine.* 2020:1-10.
101. Monfared A, Dashti-Khavidaki S, Jafari R, et al. Clinical characteristics and outcome of COVID-19 pneumonia in kidney transplant recipients in Razi hospital, Rasht, Iran. *Transplant infectious disease : an official journal of the Transplantation Society.* 2020:e13420.
102. Montastruc F, Romano C, Montastruc JL, et al. Pharmacological characteristics of patients infected with SARS-Cov-2 admitted to Intensive Care Unit in South of France. *Therapie.* 2020;75(4):381-384.
103. Montero F, Martínez-Barrio J, Serrano-Benavente B, et al. Coronavirus disease 2019 (COVID-19) in autoimmune and inflammatory conditions: clinical characteristics of poor outcomes. *Rheumatology international.* 2020;40(10):1593-1598.
104. Morena V, Milazzo L, Oreni L, et al. Off-label use of tocilizumab for the treatment of SARS-CoV-2 pneumonia in Milan, Italy. *European journal of internal medicine.* 2020;76:36-42.
105. Moreno-Pérez O, Andres M, Leon-Ramirez JM, et al. Experience with tocilizumab in severe COVID-19 pneumonia after 80 days of follow-up: A retrospective cohort study. *Journal of autoimmunity.* 2020:102523.
106. Morrison AR, Johnson JM, Griebe KM, et al. Clinical characteristics and predictors of survival in adults with coronavirus disease 2019 receiving tocilizumab. *Journal of autoimmunity.* 2020:102512.
107. Mostaza JM, García-Iglesias F, González-Alegre T, et al. Clinical course and prognostic factors of COVID-19 infection in an elderly hospitalized population. *Archives of gerontology and geriatrics.* 2020;91:104204.
108. Nguyen Y, Corre F, Honsel V, et al. A nomogram to predict the risk of unfavourable outcome in COVID-19: a retrospective cohort of 279 hospitalized patients in Paris area. *Annals of medicine.* 2020:1-9.
109. Nowak B, Szymański P, Pańkowski I, et al. Clinical characteristics and short-term outcomes of patients with coronavirus disease 2019: a retrospective single-center experience of a designated hospital in Poland. *Polish archives of internal medicine.* 2020;130(5):407-411.

110. Okoh AK, Sossou C, Dangayach NS, et al. Coronavirus disease 19 in minority populations of Newark, New Jersey. *International journal for equity in health.* 2020;19(1):93.
111. Palmieri L, Vanacore N, Donfrancesco C, et al. Clinical Characteristics of Hospitalized Individuals Dying with COVID-19 by Age Group in Italy. *The journals of gerontology Series A, Biological sciences and medical sciences.* 2020.
112. Pasquini Z, Montalti R, Temperoni C, et al. Effectiveness of remdesivir in patients with COVID-19 under mechanical ventilation in an Italian ICU. *The Journal of antimicrobial chemotherapy.* 2020.
113. Pelayo J, Lo KB, Bhargav R, et al. Clinical Characteristics and Outcomes of Community- and Hospital-Acquired Acute Kidney Injury with COVID-19 in a US Inner City Hospital System. *Cardiorenal medicine.* 2020;10(4):223-231.
114. Pellaud C, Grandmaison G, Pham Huu Thien HP, et al. Characteristics, comorbidities, 30-day outcome and in-hospital mortality of patients hospitalised with COVID-19 in a Swiss area - a retrospective cohort study. *Swiss medical weekly.* 2020;150:w20314.
115. Potere N, Di Nisio M, Rizzo G, et al. Low-Dose Subcutaneous Tocilizumab to Prevent Disease Progression in Patients with Moderate COVID-19 Pneumonia and Hyperinflammation. *International journal of infectious diseases : IJID : official publication of the International Society for Infectious Diseases.* 2020.
116. Primmaz S, Le Terrier C, Suh N, et al. Preparedness and Reorganization of Care for Coronavirus Disease 2019 Patients in a Swiss ICU: Characteristics and Outcomes of 129 Patients. *Critical care explorations.* 2020;2(8):e0173.
117. Quartuccio L, Sonaglia A, McGonagle D, et al. Profiling COVID-19 pneumonia progressing into the cytokine storm syndrome: Results from a single Italian Centre study on tocilizumab versus standard of care. *Journal of clinical virology : the official publication of the Pan American Society for Clinical Virology.* 2020;129:104444.
118. Rosenberg ES, Dufort EM, Udo T, et al. Association of Treatment With Hydroxychloroquine or Azithromycin With In-Hospital Mortality in Patients With COVID-19 in New York State. *Jama.* 2020;323(24):2493-2502.
119. Salacup G, Lo KB, Gul F, et al. Characteristics and clinical outcomes of COVID-19 patients in an underserved-inner city population: A single tertiary center cohort. *Journal of medical virology.* 2020.
120. Sentilhes L, De Marcillac F, Jouffrieau C, et al. Coronavirus disease 2019 in pregnancy was associated with maternal morbidity and preterm birth. *American journal of obstetrics and gynecology.* 2020.
121. Shah SJ, Barish PN, Prasad PA, et al. Clinical features, diagnostics, and outcomes of patients presenting with acute respiratory illness: A retrospective cohort study of patients with and without COVID-19. *EClinicalMedicine.* 2020:100518.
122. Shi D, Wu W, Wang Q, et al. Clinical Characteristics and Factors Associated With Long-Term Viral Excretion in Patients With Severe Acute Respiratory Syndrome Coronavirus 2 Infection: a Single-Center 28-Day Study. *The Journal of infectious diseases.* 2020;222(6):910-918.
123. Skipper CP, Pastick KA, Engen NW, et al. Hydroxychloroquine in nonhospitalized adults with early COVID-19: a randomized trial. 2020.
124. Spinner CD, Gottlieb RL, Criner GJ, et al. Effect of Remdesivir vs Standard Care on Clinical Status at 11 Days in Patients With Moderate COVID-19: A Randomized Clinical Trial. *Jama.* 2020.
125. Suleyman G, Fadel RA, Malette KM, et al. Clinical Characteristics and Morbidity Associated With Coronavirus Disease 2019 in a Series of Patients in Metropolitan Detroit. *JAMA Netw Open.* 2020;3(6):e2012270.
126. Tomazini BM, Maia IS, Cavalcanti AB, et al. Effect of Dexamethasone on Days Alive and Ventilator-Free in Patients With Moderate or Severe Acute Respiratory Distress Syndrome and COVID-19: The CoDEX Randomized Clinical Trial. *Jama.* 2020.
127. Toniati P, Piva S, Cattalini M, et al. Tocilizumab for the treatment of severe COVID-19 pneumonia with hyperinflammatory syndrome and acute respiratory failure: A single

center study of 100 patients in Brescia, Italy. *Autoimmunity reviews.* 2020;19(7):102568.
128. Vahidy FS, Drews AL, Masud FN, et al. Characteristics and Outcomes of COVID-19 Patients During Initial Peak and Resurgence in the Houston Metropolitan Area. *Jama.* 2020.
129. Wu J, Huang J, Zhu G, et al. Systemic corticosteroids and mortality in severe and critical COVID-19 patients in Wuhan, China. *The Journal of clinical endocrinology and metabolism.* 2020.
130. Xu P, Huang J, Fan Z, et al. Arbidol/IFN-α2b therapy for patients with corona virus disease 2019: a retrospective multicenter cohort study. *Microbes and infection.* 2020;22(4-5):200-205.
131. Yan D, Liu XY, Zhu YN, et al. Factors associated with prolonged viral shedding and impact of lopinavir/ritonavir treatment in hospitalised non-critically ill patients with SARS-CoV-2 infection. *The European respiratory journal.* 2020;56(1).
132. Yan X, Han X, Peng D, et al. Clinical Characteristics and Prognosis of 218 Patients With COVID-19: A Retrospective Study Based on Clinical Classification. *Frontiers in medicine.* 2020;7:485.
133. Zerah L, Baudouin É, Pépin M, et al. Clinical Characteristics and Outcomes of 821 Older Patients with SARS-Cov-2 Infection Admitted to Acute Care Geriatric Wards. *The journals of gerontology Series A, Biological sciences and medical sciences.* 2020.
134. Zhang Q, Wei Y, Chen M, Wan Q, Chen X. Clinical analysis of risk factors for severe COVID-19 patients with type 2 diabetes. *Journal of diabetes and its complications.* 2020:107666.
135. Zhang Y, Cui Y, Shen M, et al. Association of diabetes mellitus with disease severity and prognosis in COVID-19: A retrospective cohort study. *Diabetes research and clinical practice.* 2020;165:108227.
136. Zhu Z, Lu Z, Xu T, et al. Arbidol monotherapy is superior to lopinavir/ritonavir in treating COVID-19. 2020;81(1):e21-e23.
